# Long noncoding RNA *GAS5* disrupts intestinal epithelial barrier function by increasing small vault RNA levels

**DOI:** 10.1172/jci.insight.198593

**Published:** 2026-01-22

**Authors:** Ting-Xi Yu, Hee Kyoung Chung, Amy VanderStoep, Bridgette Warner, Hongxia Chen, Haonan Zhao, Ana S.G. Cunningham, Rosemary Kozar, Myriam Gorospe, Lan Xiao, Jian-Ying Wang

**Affiliations:** 1 Cell Biology Group, Department of Surgery, and; 2 Shock Trauma Center, University of Maryland School of Medicine, Baltimore, Maryland, USA.; 3 Laboratory of Genetics and Genomics, National Institute on Aging, NIH, Baltimore, Maryland, USA.; 4 Baltimore Veterans Affairs Medical Center, Baltimore, Maryland, USA.; 5 Department of Pathology, University of Maryland School of Medicine, Baltimore, Maryland, USA.

**Keywords:** Cell biology, Gastroenterology, Tight junctions

## Abstract

Disruptions in the integrity of the intestinal epithelium occur commonly in inflammatory bowel disease (IBD) and critical surgical disorders, but the underlying mechanisms remain largely unknown. Here we identified long noncoding RNA *GAS5* as a repressor of intestinal mucosal growth and the function of the gut epithelial barrier. The levels of tissue *GAS5*/*Gas5* increased in mouse intestinal mucosa after colitis and septic stress, as well as in human intestinal mucosa from patients with IBD. Transient and tissue-specific knockdown of *Gas5* in mice using CRISPR/Cas9 enhanced the renewal of the mucosa of the small intestine, increased the levels of tight junction (TJ) proteins ZO-1, ZO-2, claudin-1, and claudin-2, and improved gut barrier function. Conversely, ectopic overexpression of *GAS5* in intestinal organoids and in cultured intestinal epithelium cells decreased the levels of these TJ proteins and caused epithelial barrier dysfunction. Mechanistic studies revealed that *GAS5* acted as a transcriptional enhancer of the gene encoding small noncoding vault RNAs (vtRNAs) and that *GAS5* repressed TJ expression by increasing the levels of vtRNAs. Together, our results indicate that *GAS5* disrupts the integrity of the intestinal epithelium by impairing mucosal growth and epithelial barrier function and that it represses TJ expression, at least in part, via vtRNAs.

## Abstract 2

Long noncoding RNA GAS5 damages gut epithelial integrity by inhibiting mucosal growth and disrupting epithelial barrier function via interaction with vult RNAs.

## Graphical abstract

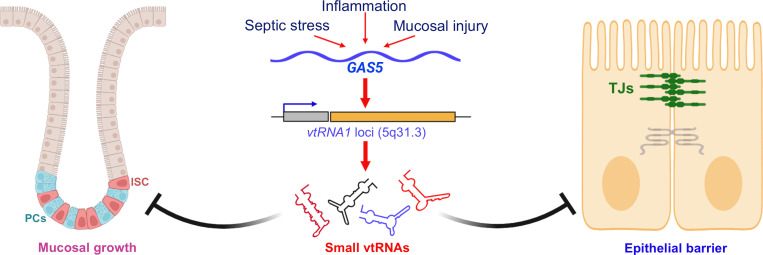

## Introduction

The epithelium of the mammalian intestinal mucosa is a single layer of columnar cells and undergoes rapid and constant self-renewal throughout the entire life of the organism (1). The intestinal stem cells (ISCs) located at the crypt base divide continuously, giving rise to proliferating progenitors that differentiate into various mature cell types when they migrate toward the intestinal lumen along the crypt-villus axis (2, 3). Differentiated intestinal epithelial cells (IECs), connected by tight junctions (TJs) and adherens junctions (AJs), establish a selectively permeable epithelial barrier that protects the subepithelial tissue of the mucosa against a wide array of luminal noxious substances, pathogens, and microbiome (3, 4). TJs are the apical-most elements of the junctional complex that seal epithelial cells together and prevent even small molecules from leaking between cells (4, 5). Immediately below the TJs are the cadherin-rich AJs that mediate strong cell-to-cell adhesion and play a critical role in regulating the epithelial barrier function (6). In response to pathophysiological stresses, maintenance of the intestinal epithelium homeostasis requires rapid changes in gene expression patterns to regulate IEC survival, proliferation, migration, differentiation, and cell-to-cell interactions (2, 3). However, the intestinal epithelium is disrupted in various human diseases, including inflammatory bowel disease (IBD), and critical surgical disorders such as sepsis and shock, leading to the translocation of harmful luminal substances and bacteria to the bloodstream and, in some cases, resulting in multiple organ dysfunction syndrome and death (7–9).

The majority of the mammalian genome (>96%) is transcribed into a large array of noncoding RNAs (ncRNAs), whereas protein-coding transcripts (mRNAs) account for only a small portion (<4%) of the expressed RNAs (10). Long ncRNAs (lncRNAs) are defined as transcripts spanning greater than 200 nucleotides in length and are expressed in tissue-, differentiation stage–, and cell type–dependent manners, with active roles in gene regulation (10, 11). Emerging evidence indicates that lncRNAs function as molecular scaffolds, decoys, or signals to regulate virtually every level of gene expression (11, 12) and that deregulation of lncRNAs is intimately involved in diverse human diseases (13–15). LncRNAs modulate a variety of cellular processes and affect pathologies by operating jointly with microRNAs (miRNAs), RNA-binding proteins (RBPs), and other molecules (12, 16–18). Several lncRNAs enriched in the gut mucosa, including *H19*, *SPRY4-IT1*, *uc.173*, *uc.230*, *Gata6*, *circHIPK3*, and *Cdr1as*, participate in controlling intestinal mucosal renewal, wound healing, and gut permeability, and play an important role in maintaining the epithelial integrity in stressful environments (12, 19). In this regard, increased levels of *H19*, the precursor of miR-675, disrupt the gut barrier function (20, 21), while *SPRY4-IT1* enhances the barrier function via interaction with RBP HuR (13). *uc.173* promotes renewal of the intestinal mucosa and enhances gut barrier function by interacting with miR-195 and miR-29b (16, 22), and *uc.230* protects IECs against apoptosis and enhances mucosal repair after injury by regulating CUG-binding protein 1 (CUGBP1) by sequestering miR-503 (23).

*GAS5*, transcribed from the growth arrest-specific 5 gene located on chromosome 1q25.1, was initially identified as a lncRNA (*Gas5*) in a mouse cell model of negative growth regulation (24, 25). Although there are small open reading frames in the body of the transcript, *GAS5* does not encode any known functional proteins (26). *GAS5* is expressed in various human tissues, including the intestinal epithelium, and plays a crucial role in the pathogenesis of different cancers by altering the cell cycle and regulating apoptosis (26–28). *GAS5* also acts as a DNA decoy for the glucocorticoid receptor to respond to growth factors (22, 29), regulates transcription of the insulin receptor gene in adipocytes (30), and functions in the sequestration of some miRNAs (26, 28). *GAS5* regulates ventricular hypertrophy by altering the SRSF4/GR axis (31), reduces cell pyroptosis in sepsis-associated renal injury (32), and modulates cellular metabolic homeostasis (33). Recently, *GAS5* was found to regulate NR2B expression by interacting with miR-23a/b, while colonic extracellular vesicles (EVs) isolated from patients with postinfectious, diarrhea-predominant, irritable bowel syndrome induce murine enteric neuroplasticity through functional interplay of *GAS5*, miR-23, and NMDA NR2b (34). To date, however, no studies have investigated the role of *GAS5* in regulating the constitutive renewal of the intestinal mucosa and gut barrier function.

In this study, we provide evidence that *GAS5* suppresses the homeostasis of the intestinal epithelium. Our results show that tissue levels of *GAS5/Gas5* in the intestinal mucosa increase markedly in patients with IBD and in mice experiencing dextran sodium sulfate–induced (DSS-induced) colitis and septic stress–induced gut barrier dysfunction, and that decreasing the levels of cellular *GAS5* promotes growth of the intestinal mucosa and enhances epithelial barrier function. We also present evidence that *GAS5* inhibits TJ expression by increasing the levels of small noncoding vault RNAs (vtRNAs), a class of small ncRNAs involved in many cellular processes. These findings demonstrate the importance of *GAS5* and vtRNAs in maintaining the integrity of the intestinal epithelium and point to *GAS5* and vtRNAs as potential therapeutic targets for interventions to promote mucosal growth and protect gut barrier function in patients with critical diseases.

## Results

### Changes in GAS5/Gas5 levels in the intestinal epithelium responding to stress.

**Figure 1:**
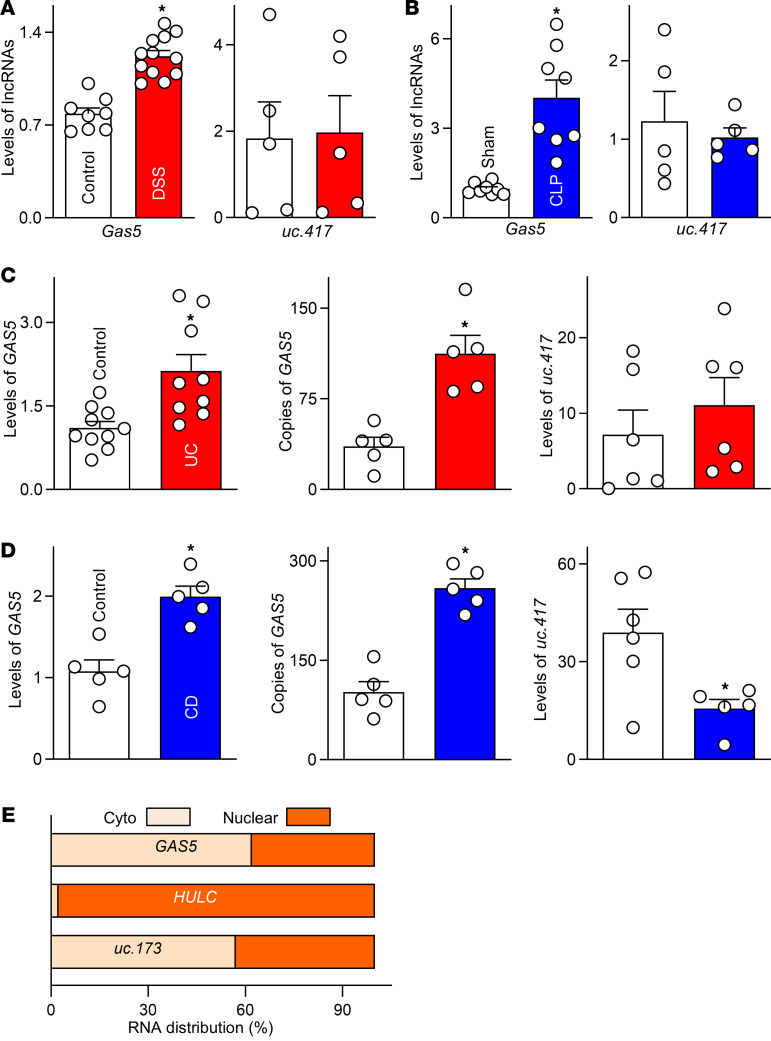
Mucosal *GAS5/Gas5* expression in the intestine associated with various pathologies. (**A**) Levels of mucosal *Gas5* and *uc.417* in the colon from mice treated with water (control) or 3% DSS in drinking water for 7 days, as measured using RT-qPCR analysis. Values are the mean ± SEM (*n* = 8 or 12). *P < 0.05 compared with control. (**B**) Levels of mucosal *Gas5* and *uc.417* in the small intestine of mice exposed to CLP for 48 hours (*n* = 5 or 8). *P < 0.05 compared with sham. (**C**) Levels of mucosal *GAS5* (left), copy number as measured by ddPCR analysis (middle), and *uc.417* (right) in the colon from patients with ulcerative colitis (UC). Values are the mean ± SEM (*n* = 5–10). *P < 0.05 compared with controls. (**D**) Levels of mucosal *GAS5* levels (left), its copy number (middle), and *uc.417* (right) in the ileum from patients with Crohn’s disease (CD) (*n* = 5 or 6). *P < 0.05 compared with controls. (**E**) Levels of cytoplasmic (Cyto) and nuclear lncRNAs *GAS5*, *HULC*, and *uc.173* in Caco-2 cells. In **A**–**D**, statistical significance was analyzed using unpaired, 2-tailed Student’s *t* test. In **E**, experiments were repeated 3 times with similar results.

To determine the involvement of *Gas5* in the intestinal epithelium homeostasis in stressful environments, 2 murine models, DSS-induced mucosal inflammatory injury in colon (35) and cecal ligation and puncture–induced (CLP-induced) mucosal injury in small intestine and gut barrier dysfunction (36), were used in this study. Consistent with our previous work and other studies (23, 35, 37), after administration of 3% DSS in drinking water for 7 days, mice exhibited injury/erosions, granulocyte infiltration in the colonic mucosa (Supplemental Figure 1A; supplemental material available online with this article; https://doi.org/10.1172/jci.insight.198593DS1), and bloody diarrhea, mimicking the damages observed in human ulcerative colitis (UC). As shown in Figure 1A, the levels of mucosal *Gas5* increased significantly in the colons of DSS-treated mice, achieving greater than 1.7-fold higher levels than those observed in control animals. This increase in mucosal *Gas5* abundance was specific, since treatment with DSS for 7 days did not alter the colon mucosal levels of lncNA *uc.417* that is also highly expressed in the intestinal epithelium. Microscopic examination revealed that exposure of mice to CLP for 48 hours induced mucosal lesions in the small intestine, denuded villi with dilated capillaries, hemorrhage, and sloughed cells (Supplemental Figure 1A), and macroscopic examination revealed edematous and swollen mucosa with areas of red streaks, as reported previously (15, 38). Exposure to CLP for 48 hours also resulted in an acute gut barrier dysfunction, as evidenced by an increased mucosa permeability to FITC-dextran, as reported previously (38). In CLP mice, induced mucosal injury and gut barrier dysfunction were also associated with a substantial increase in the levels of mucosal *Gas5* in the small intestine without affecting the abundance of *uc.417* (Figure 1B). The latter finding again indicates the specificity of the increase in mucosal *Gas5* in response to pathological stress in the small intestine.

To begin to understand whether possible changes in *GAS5* levels might be clinically relevant, we examined the levels of *GAS5* in human intestinal mucosal tissues from patients with IBD. Colonic mucosal tissues from patients with UC and small intestinal mucosa from patients with Crohn’s disease (CD) were collected and the levels of *GAS5* and *uc.417* were measured. Mucosal tissue from patients without injury/erosions, inflammation, or gut barrier dysfunction served as controls. Interestingly, intestinal mucosal tissues from both UC and CD patients exhibited increased levels of *GAS5*, as measured by reverse transcription (RT) followed by quantitative PCR (qPCR) analysis (Figure 1, C and D). Droplet digital PCR (ddPCR) analysis further showed that copy numbers of *GAS5* in the mucosa increased markedly in patients with UC and CD relative to controls (Figure 1, C and D). In this study, *GAS5* was read from at least 10,000 droplets in each well of the mucosal samples for absolute quantification. Consistent with the findings observed in DSS-induced colitis in mice, there were no significant changes in the levels of *uc.417* in the colonic mucosa between patients with UC and controls (Figure 1C), but the mucosa from patients with CD displayed decreased abundances of *uc.417* in the small intestine (Figure 1D). The increased levels of the mucosal *GAS5* in patients with UC and CD were associated with severe mucosal injury/erosions, inflammation, delayed healing, and gut barrier dysfunction; we previously reported similar findings (15, 39, 40). In addition, *GAS5* was found in both the cytoplasm and the nucleus of cultured human IECs (Figure 1E), similar to the distribution of the lncRNA *uc.173*. In contrast, lncRNA *HULC* was predominantly localized in the nucleus, as reported previously (16). Together, the results obtained from experiments in mouse and human intestinal mucosa indicate that expression levels of tissue *GAS5* are dramatically affected in response to various critical stresses and strongly suggest that *GAS5* influences the homeostasis of the intestinal epithelium and may play a role in human gut mucosal pathologies.

### GAS5 inhibits renewal of the intestinal epithelium and causes gut barrier dysfunction.

**Figure 2:**
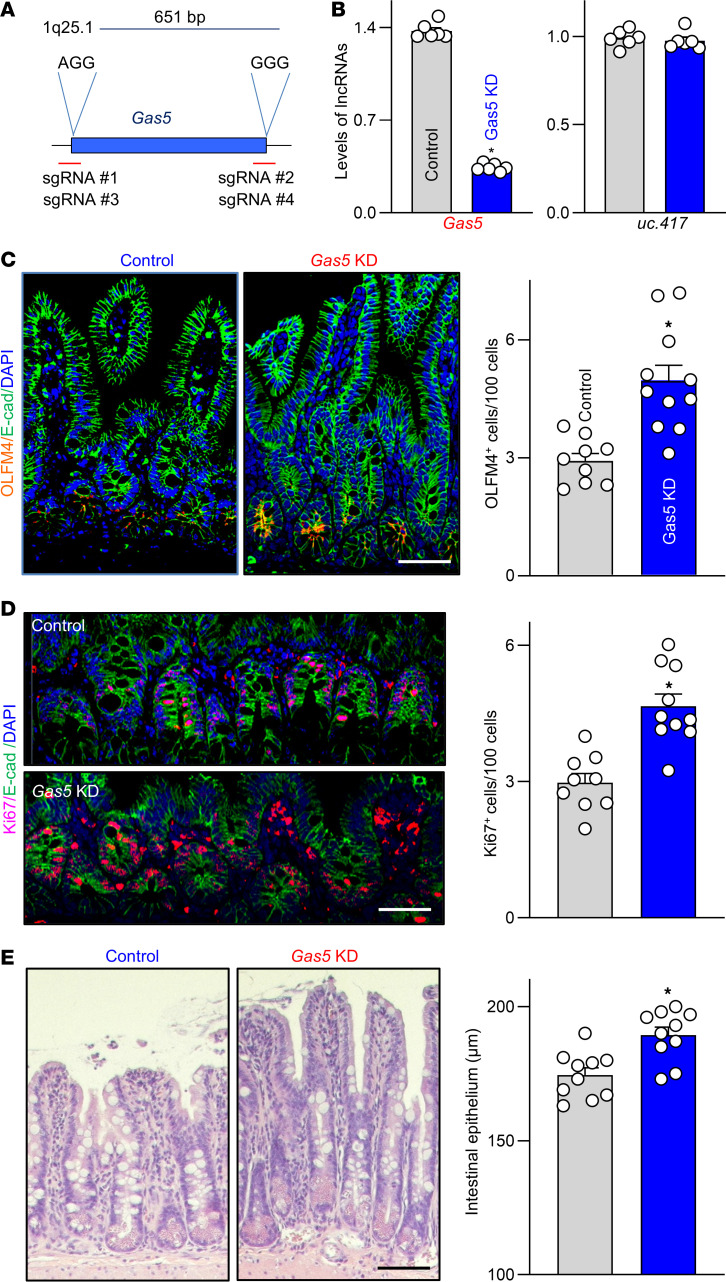
Targeted deletion of the *Gas5* by CRISPR/Cas9 in mice enhances renewal of the small intestinal epithelium. (**A**) The sequences given denote protospacer-adjacent motifs. sgRNA, small guide RNA. (**B**) Levels of *Gas5* and *uc.417* in the small intestinal mucosa in control and *Gas5*-knockdown (*Gas5*-KD) mice, as measured by RT-qPCR analysis. IE-Cas9 mice were injected i.p. with AVV-sgRNA-*Gas5* or AAV-Con, and the levels of tissue *Gas5* and *uc.417* in the small intestinal mucosa of IE-Cas9 mice were examined on day 7 after the injection. Values are the mean ± SEM (*n* = 6). *P < 0.05 compared with controls. (**C** and **D**) Proliferating cells in the small intestinal mucosa of control and *Gas5*-KD mice, as measured by immunostaining of OLFM4 and Ki67. Orange, OLFM4; pink, Ki67; and green, E-cadherin (E-cad). (**E**) Photomicrographs of H&E (left) and changes in the length of the epithelium (right) of mucosa described in **B**. Values are the mean ± SEM (*n* = 10). *P < 0.05 compared with controls. Scale bars: 50 μm. In **B** and **E**, statistical significance was analyzed using unpaired, 2-tailed Student’s *t* test. In **C** and **D**, all experiments were repeated 3 times with similar results.

To examine the in vivo function of *GAS5* in the intestine, we used CRISPR/Cas9-knockin mice to specifically knockdown *Gas5* in the intestinal epithelium by overexpressing specific small guide RNAs (sgRNAs) directed at the *Gas5* gene (sgRNA-*Gas5*) (Figure 2A), as described previously (41, 42). Intestinal epithelium–specific, Cre-dependent, and constitutive Cas9-expressing (IE-Cas9) mice were generated by crossing Cre-dependent Cas9 mice with villin-Cre mice (42). The sgRNA-*Gas5* and control oligonucleotides were designed, synthesized, and selected in cultured cells as targeting the *Gas5* locus with maximal efficiency and minimal off-target effects before studies using mice. In cultured HCT116-Cas9 cells, transfection with specific sgRNAs directed at *Gas5* (Supplemental Figure 1B), including sgRNA-*Gas5* and sgRNA-*Gas5*_2 (sgRNA-Gas5-A) or sgRNA-*Gas5*_3 and sgRNA-*Gas5*_4 (sgRNA-*Gas5*-B), decreased *Gas5* levels by more than 90% without effect on other lncRNAs, including *uc.417*. To gain support that the effects were mediated via direct genome editing of *Gas5* in IE-Cas9 mice, we used adeno-associated virus–mediated (AAV-mediated) delivery of sgRNA-*Gas5* and constructed the AAV-sgRNA vectors containing either sgRNA-*Gas5*-A (AAV-sgRNA-*Gas5*-A) or sgRNA-*Gas5*-B (AAV-sgRNA-*Gas5*-B), while AAV vector containing no sgRNA (AAV-Con) served as control.

Age-matched IE-Cas9 mice were injected intraperitoneally (i.p.) with both AAV-sgRNA-*Gas5*-A and AAV-sgRNA-*Gas5*-B or AAV-Con. RT-qPCR analysis showed that *Gas5* levels decreased dramatically in the small intestinal mucosa of IE-Cas9 mice on day 7 after injection with AAV-sgRNA-*Gas5* (Figure 2B), although *uc.417* expression levels were normal (Figure 2B). On the other hand, there were no changes in the levels of *Gas5* in kidney, spleen, stomach, or liver between mice infected with AAV-sgRNA-*Gas5* and mice infected with AAV-Con (Supplemental Figure 1C). This intestinal epithelium–specific knockdown of *Gas5* in IE-Cas9 mice was transient and was not transmitted to the next generation, as reported previously (41, 43); these newly generated conditional *Gas5*-knockdown (*Gas5*-KD) mice were used in subsequent experiments.

**Figure 3:**
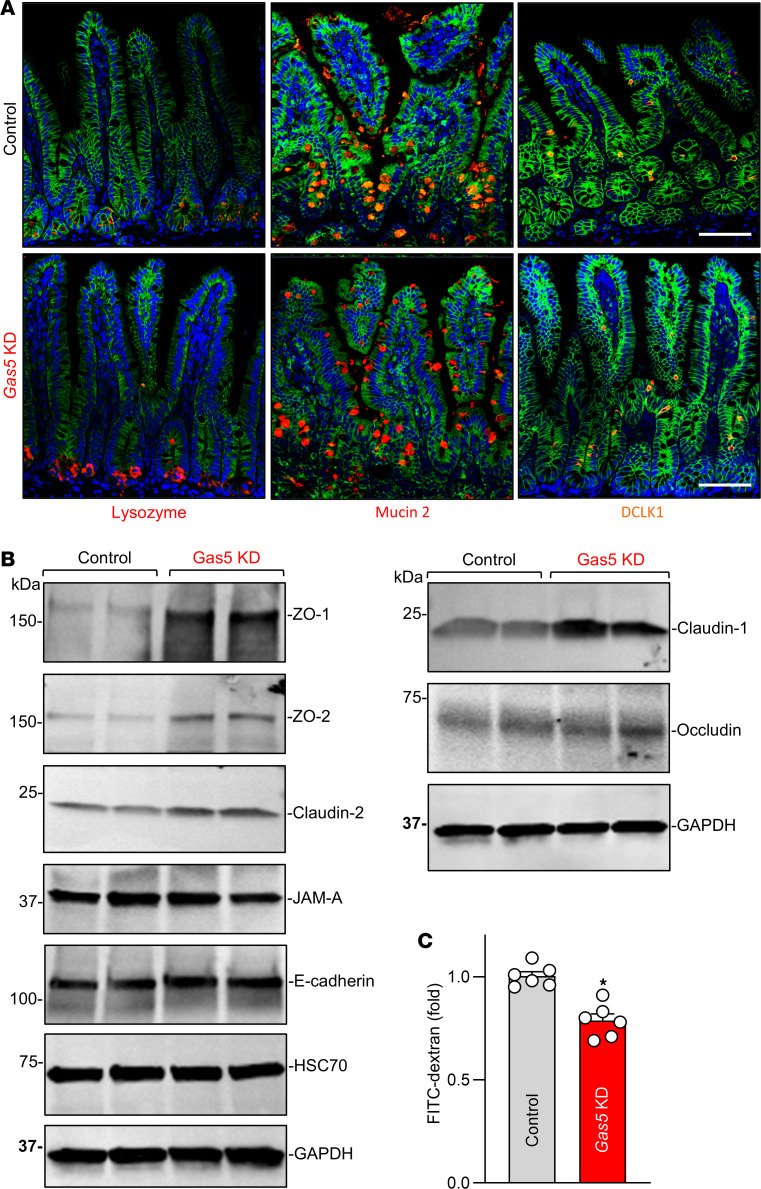
Decreasing the levels of *Gas5* increases Paneth cells and improves gut barrier function. (**A**) Immunostaining of Paneth (lysozyme-positive), goblet (mucin 2–positive), and tuft (DCLK1-positive) cells in the small intestinal mucosa of control and *Gas5*-KD mice. Red, lysozyme, mucin 2, or DCLK1. Green, E-cadherin. Scale bars: 50 μm. (**B**) Immunoblots of intercellular junction proteins in the small intestinal mucosa of control and *Gas5*-KD mice. Total proteins were isolated from the mucosal tissues and prepared for Western blot analysis. Equal loading was monitored by GAPDH. (**C**) Changes in gut permeability in control and *Gas5*-KD mice. FITC-dextran was given orally, and blood samples were collected 4 hours later for measurement. Values are the mean ± SEM (*n* = 6). *P < 0.05 compared with control. In **C**, statistical significance was analyzed using unpaired, 2-tailed Student’s *t* test. In **A** and **B**, 3 separate experiments were performed and showed similar results.

Decreasing the levels of tissue *Gas5* in IE-Cas9 mice by ablating the *Gas5* locus from the mouse genome with AAV-sgRNA-*Gas5* promoted a robust renewal of the small intestinal epithelium. The intestinal mucosa from *Gas5*-KD mice exhibited increased activity of stem cells, as marked by OLFM4 immunostaining (Figure 2C), induction in the proliferating crypt cell population as shown by increased Ki67 immunostaining (Figure 2D), and longer villi and crypts (Figure 2E). The lengths of the small intestinal epithelium in *Gas5*-KD mice increased by approximately 20% when compared with those observed in mice infected with AAV-Con. Decreasing the levels of tissue *Gas5* also enhanced the function of Paneth cells in the intestinal mucosa (Figure 3A and Supplemental Figure 2A), but failed to alter the levels of goblet cells (Figure 3A) or tuft cells (Figure 3A). Staining whole mounts of the small intestine showed that lysozyme-positive cells (Paneth cells) were located at the base of the crypt areas, but mucin 2–positive cells (goblet cells) and double cortin–like kinase 1–positive (DCLK1-positive) cells (tuft cells) were distributed at both villous and crypt regions. The numbers of Paneth cells in the small intestinal mucosa were approximately 2-fold higher in *Gas5*-KD mice relative to control mice. However, there were no significant differences in the numbers of goblet cells and tuft cells in the small intestinal mucosa between *Gas5*-KD mice and controls. In addition, *Gas5* deletion did not affect enterocyte differentiation in the small intestinal mucosa, as determined by villin immunostaining analysis (Supplemental Figure 2B).

The *Gas5*-deficient mucosa of the small intestine also exhibited increased levels of TJ proteins ZO-1, ZO-2, claudin-1, and claudin-2, but not occludin, JAM-A, AJ protein E-cadherin, and heat shock protein HSC70 (Figure 3B and Supplemental Figure 2C). Consistent with an increase in the levels of these TJ proteins, targeted deletion of the *Gas5* locus in the intestinal epithelium by infection with AAV-sgRNA-*Gas5* also improved gut barrier function, since *Gas5*-KD mice exhibited a lower basal level of gut permeability than that observed in mice infected with AAV-Con (Figure 3C), as examined by FITC-dextran assays (36, 38).

**Figure 4:**
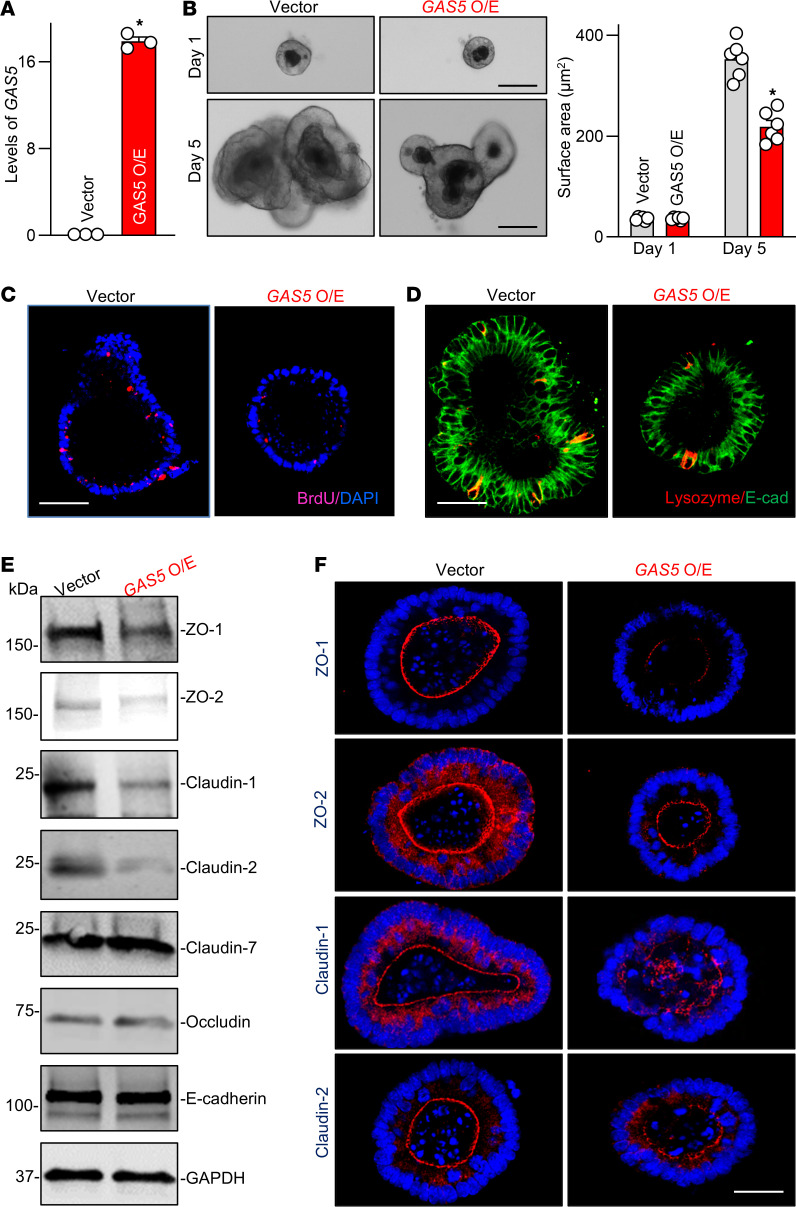
Ectopic overexpression of *Gas5* inhibits enteroid growth and decreases tight junction proteins ex vivo in organoids. (**A**) Levels of *Gas5* in enteroids 48 hours after transfection with *Gas5* overexpression vector. Values are the mean ± SEM (*n* = 3). *P < 0.05 compared with control vector. (**B**) Growth inhibition of enteroids by *Gas5* overexpression. Enteroids were derived from the proximal small intestine of wild-type mice and transfected with the *Gas5* expression vector on day 1 after primary culture. Images were taken on day 5 after the transfection. Values are the mean ± SEM (*n* = 6). *P < 0.05 compared with control vector. (**C** and **D**) BrdU labeling and immunostaining of lysozyme (marker for Paneth cells) in enteroids on day 3 after transfection. (**E** and **F**) Immunoblots and immunostaining of intercellular junctions in enteroids treated as described in **C**. Scale bars: 50 μm. In **A** and **B**, statistical significance was analyzed using unpaired, 2-tailed Student’s *t* test. In all other studies, experiments were repeated 3 times and showed similar results.

To define the potential implication of factors secreted by stromal or immune cells of the gut in modulating the intestinal mucosa growth and TJ expression after *Gas5* deletion, intestinal organoids were derived from proliferating crypts of mouse small intestinal mucosa. Intestinal organoids were initially grown from tiny proliferating crypts, but after 5 days in culture, the organoids consisted of multiple cells and buds. As shown in Figure 4A, the levels of cellular *Gas5* increased dramatically by transfection with the plasmid expressing *Gas5* under control of the pCMV promoter (pcDNA3.1 backbone); by 48 hours later, *Gas5* levels were strongly elevated, while other lncRNAs such as *uc.417* were not (data not shown). Ectopically expressing *Gas5* markedly reduced the sizes of organoids and inhibited DNA synthesis in multiple cells, as indicated by a reduction in their surface areas (Figure 4B) and a decrease in BrdU incorporation (Figure 4C and Supplemental Figure 3A) in the organoids transfected with the *Gas5* expression vector, compared with organoids transfected with control vector. Moreover, *Gas5* overexpression led to defects in Paneth cells in intestinal organoids, as evidenced by decreased numbers of lysozyme-positive cells after transfection with the *Gas5* expression vector (Figure 4D and Supplemental Figure 3A).

Increasing the levels of *Gas5* in intestinal organoids also decreased the levels of TJ proteins ZO-1, ZO-2, claudin-1, and claudin-2 without affecting the levels of occludin, claudin-7, or E-cadherin (Figure 4E and Supplemental Figure 3B), as measured by Western blot analysis. Immunofluorescence microscopy revealed that in the control group, ZO-1 was limited to or near the apical membrane (Figure 4F), while ZO-2 and claudin-1 lined the apical membrane and basolateral regions, with slight cytoplasmic staining present (Figure 4F). The protein claudin-2 predominantly lined the apical region with slight cytoplasmic staining present in some organoids (Figure 4F). Consistent with this pattern of expression, there were significant decreases in the levels of staining intensity of ZO-1, ZO-2, claudin-1, and claudin-2 in organoids overexpressing *Gas5*, compared with those transfected with the control vector (Figure 4F and Supplemental Figure 3C). In contrast, *Gas5* overexpression did not alter the subcellular distribution of these TJ proteins in intestinal organoids. Together, the results from experiments in mice and organoids support the notion that *Gas5* is a negative regulator of the intestinal epithelium homeostasis, and its induction disrupts the integrity of the epithelium by inhibiting the constitutive growth of the mucosa and compromising the function of the epithelial barrier.

### GAS5 lowers TJ expression and increases epithelial paracellular permeability in cultured IECs.

**Figure 5:**
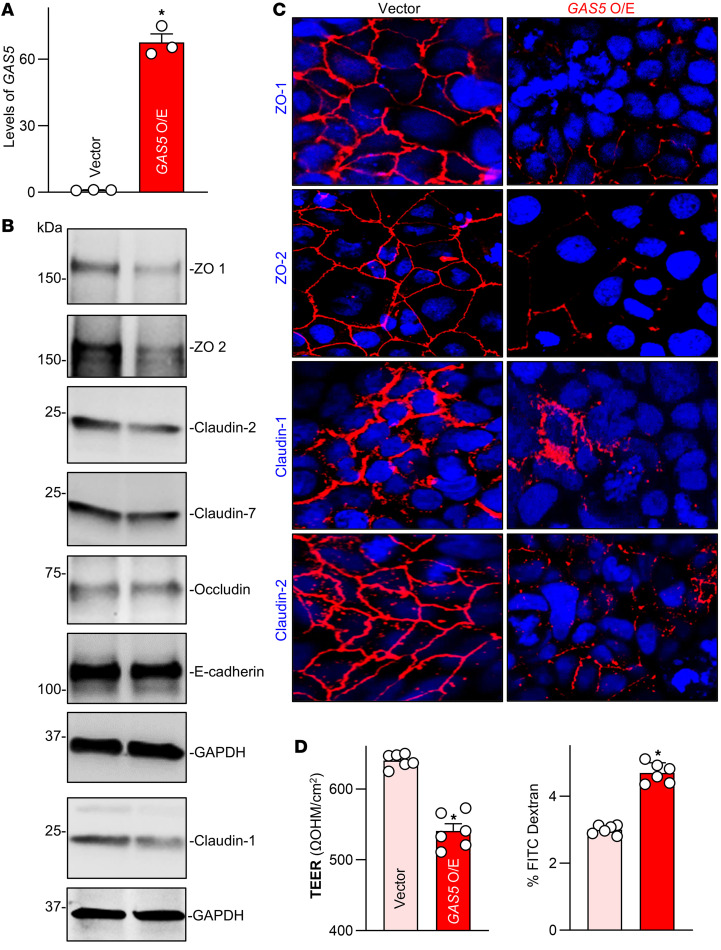
Ectopically expressed *GAS5* disrupts intestinal epithelial barrier function in cultured IECs. (**A**) Levels of *GAS5* in Caco-2 cells 48 hours after transfection with *GAS5* expression vector. Values are the mean ± SEM (*n* = 3). *P < 0.05 compared with control vector. (**B**) Expression levels of intercellular junction proteins in cells treated described in **A**, as assessed by Western blot analysis. GAPDH was included as a loading control. (**C**) Distribution of tight junction proteins in cells treated as described in **A**. Forty-eight hours after transfection, cells were fixed, permeabilized, and incubated first with antibodies against different intercellular junction proteins and then with TRITC-conjugated anti-IgG. Original magnification, ×500. (**D**) Changes in TEER (left) and FITC-dextran paracellular permeability (right) in cells treated as described in **A**. TEER assays were performed on 12-mm Transwell filters; paracellular permeability was assayed by adding the membrane-impermeable trace molecule FITC-dextran to the insert medium. Values are the mean ± SEM (*n* = 6). *P < 0.05 compared with control vector. In **A** and **D**, statistical significance was analyzed using unpaired, 2-tailed Student’s *t* test. In **B** and **C**, 3 separate experiments were performed and showed similar results.

To further examine the role of altered *GAS5* in the regulation of intestinal epithelial barrier function, 2 sets of studies were carried out in culture using human Caco-2 cells, as described previously (44, 45). First, we examined the impact of increasing the levels of cellular *GAS5* on the expression of TJs and epithelial barrier function. Ectopic overexpression of *GAS5* (Figure 5A) by transfection with its expression vector specifically decreased the levels of ZO-1, ZO-2, claudin-1, and claudin-2 proteins, but it failed to alter the expression of claudin-7, occludin, and E-cadherin (Figure 5B and Supplemental Figure 4A). Immunostaining analysis showed that ZO-1, ZO-2, claudin-1, and claudin-2 were predominantly localized on the plasma membrane in control cells but their immunostaining intensity decreased remarkably after ectopic *GAS5* overexpression (Figure 5C and Supplemental Figure 4B). Consistent with this pattern of expression, increasing the levels of cellular *GAS5* disrupted the epithelial barrier function, as evidenced by a decrease in transepithelial electrical resistance (TEER) values (Figure 5D) and an increase in the levels of paracellular flux of FITC-dextran (Figure 5D).

**Figure 6:**
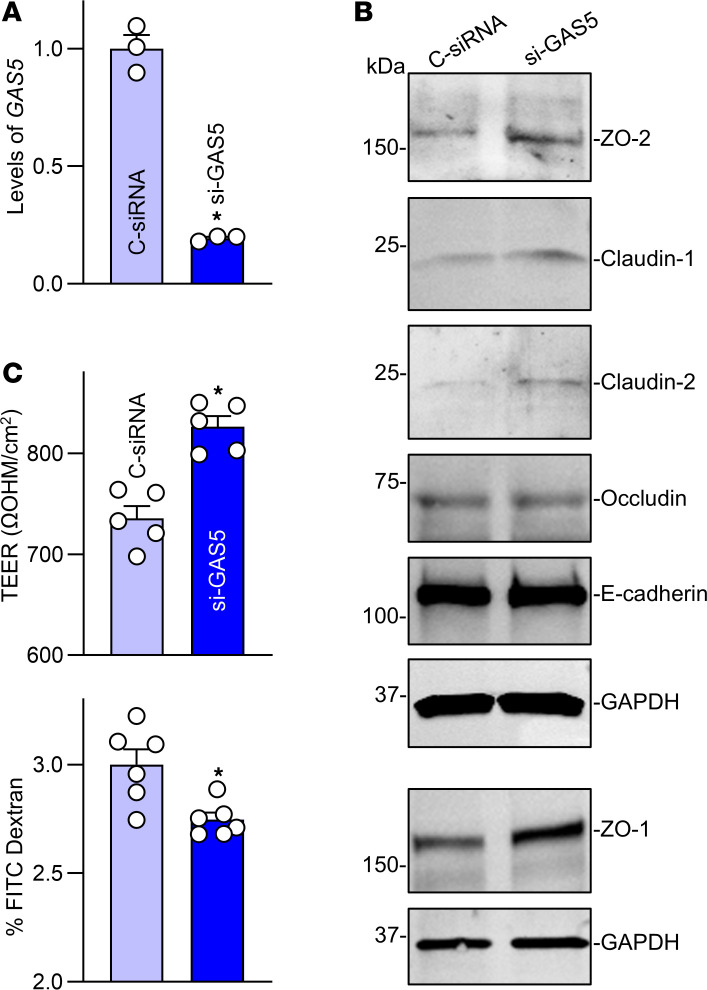
*GAS5* silencing improves intestinal epithelial barrier function in culture. (**A**) Levels of *GAS5* in Caco-2 cells 48 hours after transfection with the siRNA targeting *GAS5* (si-GAS5) or a control siRNA (C-siRNA). Values are the mean ± SEM (*n* = 3). *P < 0.05 compared with C-siRNA. (**B**) Immunoblots of intercellular junction proteins in cells treated as described in **A**. GAPDH immunoblotting served as an internal control for equal loading. (**C**) Changes in TEER (top) and FITC-dextran paracellular permeability (bottom) in cells treated as described in **A**. Values are the mean ± SEM (*n* = 6). In **A** and **C**, statistical significance was analyzed using unpaired, 2-tailed Student’s *t* test. In **B**, 3 separate experiments were performed and showed similar results.

Second, we examined the effect of *GAS5* silencing on TJ expression and epithelial barrier function in culture. As shown in Figure 6A, the levels of cellular *GAS5* decreased dramatically 48 hours after transfection with siRNA targeting *GAS5* (si-GAS5) relative to cells transfected with control siRNA (C-siRNA); no differences were seen for *uc.417* between the 2 transfection groups. The decrease in *GAS5* levels by si-GAS5 transfection increased the levels of ZO-1, ZO-2, claudin-1, and claudin-2 proteins (Figure 6B and Supplemental Figure 5A), although occludin and E-cadherin were unchanged. Moreover, *GAS5* silencing enhanced the epithelial barrier function, since it increased TEER (Figure 6C) and decreased paracellular flux of FITC-dextran (Figure 6C). These results indicate that *GAS5* inhibits TJ expression, thus contributing to dysfunction of the epithelial barrier.

### GAS5 inhibits TJs by increasing small noncoding vtRNAs.

**Figure 7:**
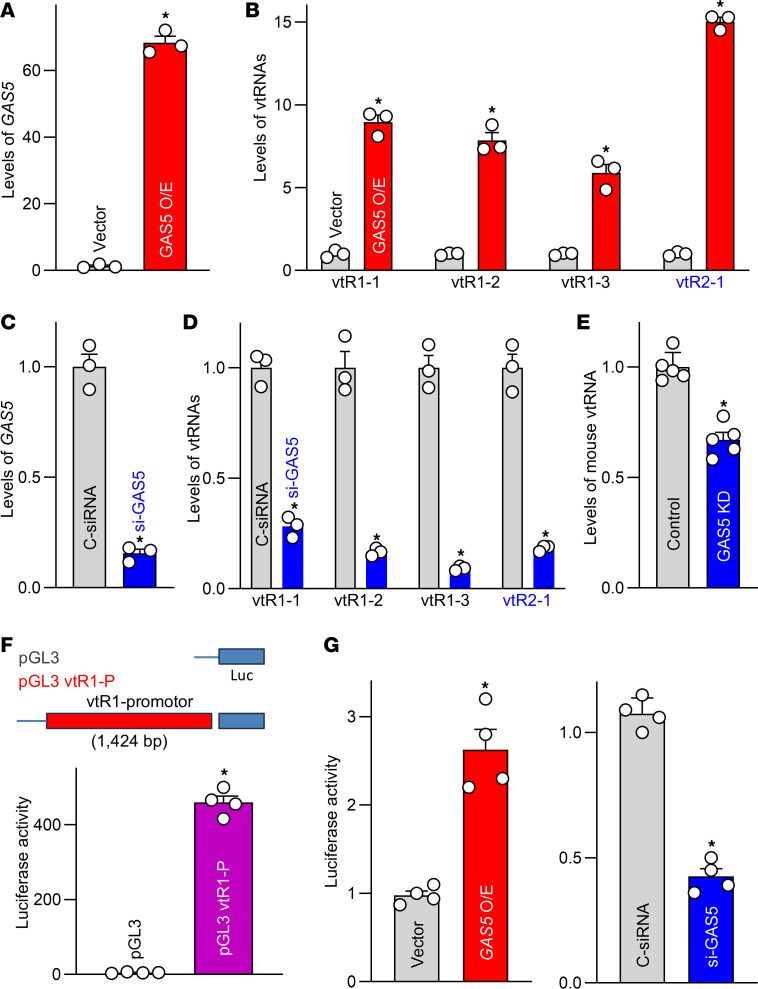
*GAS5* elevates the abundance of vtRNAs by increasing gene transcription. (**A**) Levels of *GAS5* in Caco-2 cells 48 hours after transfection with *GAS5* expression vector. Values are the mean ± SEM (*n* = 3). *P < 0.05 compared with control vector. (**B**) Changes in the levels of *vtRNA1-1* (*vtR1-1*), *vtR1-2*, *vtR1-3*, and *vtR2-1* in cells treated as described in **A** (*n* = 3). *P < 0.05 compared with control vector. (**C**) Levels of *GAS5* in Caco-2 cells 48 hours after transfection with si-GAS5 or C-siRNA. *P < 0.05 compared with C-siRNA (*n* = 3). (**D**) Levels of vtRNAs in cells treated as described in **C**. *P < 0.05 compared with C-siRNA (*n* = 3). (**E**) Levels of mouse vtRNA in the small intestinal mucosa of control and *Gas5*-knockdown (*Gas5*-KD) mice. *P < 0.05 compared with controls (*n* = 5). (**F**) Structure (top) and activity (bottom) of luciferase (Luc) reporter of the *vtRNA1* promoter. Cells were transfected with control pGL3 vector or Luc-vtRNA1 (vtR1) promoter, and the luciferase activity was examined 48 hours later. *P < 0.05 compared with pGL3 (*n* = 4). (**G**) Changes in the levels of luciferase activity of the vtR1 promoter in cells overexpressing *GAS5* (left) or *GAS5-*silent cells (right). The luciferase activity was examined 48 hours after cotransfection with GAS5 expression vector and Luc-vtR1-promoter or cotransfection with si-GAS5 and Luc-vtR1-promoter. *P < 0.05 compared with vector or C-siRNA (*n* = 4). In all studies, 2-tailed Student’s *t* test was used for statistical analysis.

To investigate the mechanism underlying *GAS5* in the regulation of gut barrier function, we tested the possibility that *GAS5* represses TJs by altering the expression of vtRNAs, a family of small (~100 nt) ncRNAs recently shown to disrupt gut barrier function via interaction with RBPs (37, 49–51). First, we determined whether altering the levels of cellular *GAS5* leads to changes in the abundance of vtRNAs. Ectopic overexpression of *GAS5* (Figure 7A) in Caco-2 cells increased the levels of all 4 human vtRNAs, *vtRNA1-1*, *vtRNA1-2*, *vtRNA1-3*, and *vtRNA2-1* (Figure 7B) between approximately 5.8 and approximately 14.5-fold, respectively, compared with cells transfected with control vector. Conversely, *GAS5* silencing (Figure 7C) by transfection with si-GAS5 decreased the levels of all 4 vtRNAs (Figure 7D) by approximately 73% to approximately 91% relative to control cells. Interestingly, the levels of endogenous mouse vtRNA also decreased in the small intestinal mucosa of *Gas5*-KD mice relative to controls (Figure 7E). Unlike humans, mice only express one vtRNA (*Vaultrc5*, or *Mvg1*), that has some degree of sequence conservation with human *vtRNA1-1* (47). These results indicate that *GAS5* is a potent stimulator of vtRNA expression.

Second, we examined the mechanism by which *GAS5* induced expression of vtRNAs in IECs. The 4 human vtRNAs are encoded on chromosome 5q31 in 2 loci; the *vtRNA-1* locus contains genetic information for *vtRNA1-1*, *vtRNA1-2*, and *vtRNA1-3*, while the *vtRNA2-1* locus encodes *vtR2-1* only (47, 48). We constructed a luciferase reporter containing the full-length *vtRNA-1* promoter (Figure 7F) and found that overexpressing *GAS5* increased the activity of the *vtRNA-1* promoter (Figure 7G), while silencing *GAS5* inhibited the activity of the *vtRNA-1* promoter (Figure 7G). Analysis of the effect of *GAS5* on vtRNA turnover revealed that ectopic overexpression of *GAS5* did not alter the stability of vtRNAs. These results suggest that *GAS5* increases the levels of *vtRNA1-1*, *vtRNA1-2*, and *vtRNA1-3* in IECs primarily by enhancing transcription.

**Figure 8:**
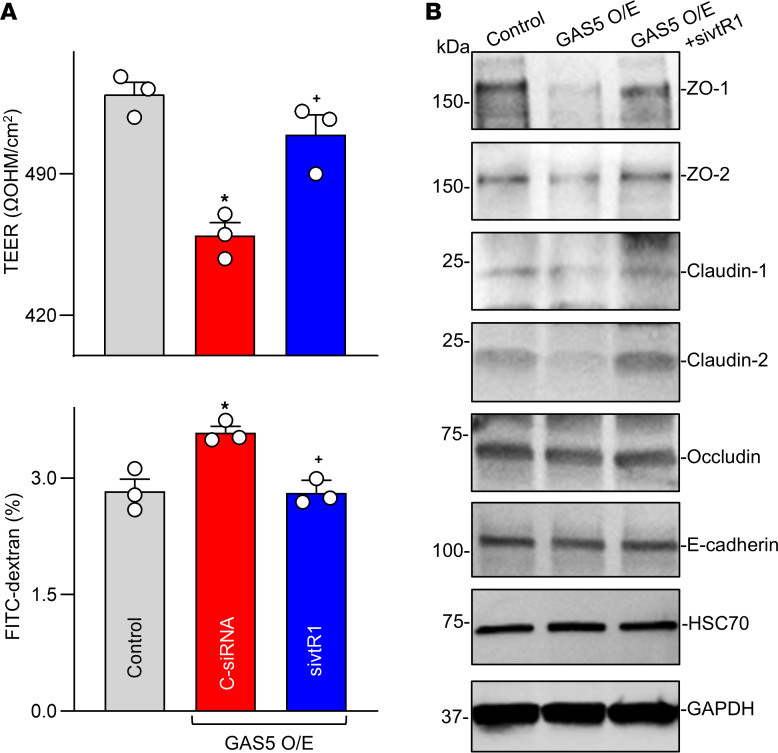
*vtRNA1-1* silencing prevents *GAS5*-induced epithelial barrier dysfunction in cultured Caco-2 cells. (**A**) Changes in TEER (top) and FITC-dextran paracellular permeability (bottom) in cells transfected with *GAS5* expression vector alone or cotransfected with the *GAS5* expression vector and siRNA targeting *vtRNA1-1* (sivtR1). TEER and permeability were examined 48 hours after transfection. Values are the mean ± SEM (*n* = 3). *P < 0.05 compared with control vector. ^+^*P* < 0.05 compared with cells cotransfected with the *GAS5* expression vector and C-siRNA. (**B**) Western blot analysis of tight junction proteins in cells treated as described in **A**; GAPDH was included as an internal loading control. In **A**, statistical significance was analyzed using 1-way ANOVA with Tukey’s post hoc test. In **B**, 3 separate experiments were performed and showed similar results.

Third, we tested whether the changes in vtRNAs elicited by *GAS5* played a role in regulating epithelial barrier function in culture. As expected, ectopically overexpressed *GAS5* impaired the epithelial barrier function, with decreased TEER and increased paracellular flux of FITC-dextran (Figure 8A) compared with control cells. However, this disruption of epithelial barrier function by *GAS5* was prevented by decreasing the levels of *vtRNA1-1* via transfection with specific siRNAs (sivtR1), as reported previously (46). There were no significant differences in TEER or paracellular flux of FITC-dextran between cells cotransfected with the *GAS5* vector and sivtR1 and cells transfected with control vector. In keeping with these findings, the loss of TJ proteins ZO-1, ZO-2, claudin-1, and claudin-2 (Figure 8B) by *GAS5* overexpression was rescued by silencing *vtRNA1-1*, with restoration of the levels of these TJs in cells cotransfected with *GAS5* expression vector and sivtR1 (Figure 8B and Supplemental Figure 5B). Overexpression of *GAS5*, alone or after cotransfection with sivtR1, did not affect the levels of occludin, E-cadherin, or HSP70. Because vtRNAs are potent biological repressors of gut epithelial homeostasis and their expression levels are tightly regulated (19, 46, 49), these findings strongly suggest that increased *GAS5* disrupts the intestinal barrier function at least partially by increasing vtRNA levels, specifically from the *vtR-1* locus.

## Discussion

The lncRNA *GAS5* is involved in many cell processes relevant to human pathologies (26, 31, 50), but its exact role in gut mucosal physiology and adaptation is underexplored. In this study, we identified *GAS5* as a biological regulator of the intestinal epithelium homeostasis by modulating mucosal growth and epithelial barrier function and found that *GAS5* expression levels in the mucosa changed markedly in response to various pathophysiological stresses. Transient and tissue-specific deletion of *Gas5* in the intestinal epithelium in CRISPR/Cas9-knockin mice not only enhanced the renewal of the intestinal mucosa but also promoted epithelial barrier function. Experiments aimed at defining the mechanism underlying the action of *GAS5* in this process revealed that ectopically expressed *GAS5* in human IECs decreased the levels of TJ proteins by increasing vtRNA expression. These findings expand our knowledge of the biological functions of *GAS5* in the intestinal epithelium and represent a new conceptual advance linking *GAS5* with intestinal mucosa growth and epithelial barrier function. Because the levels of *GAS5* increased in human intestinal mucosal tissues with injury/erosions and inflammation in patients with IBD and in septic mice with gut barrier dysfunction, our results suggest that a dysregulated *GAS5*/vtRNA paradigm plays an important role in the pathogenesis of impaired mucosal renewal, delayed repair, and gut barrier dysfunction in patients with critical illnesses.

The results reported here provide the first evidence to our knowledge of the biological role of *GAS5* in maintaining intestinal epithelium homeostasis. Consistent with our recent study (42) and other studies using CRISPR/Cas9-knockin mice (43, 51), a single injection of AAV-sgRNA-*Gas5* in IE-Cas9 mice dramatically decreased the levels of mucosal *Gas5* in the intestine only, but it failed to alter *Gas5* expression in other tissues and organs. These results further underscore the value and application of tissue-specific CRISPR/Cas9-knockin mice used in conjunction with sgRNA-*Gas5* to generate loss of function by genome editing (41–43, 51). Importantly, the transient and conditional knockdown of *Gas5* by infecting IE-Cas9 mice with AAV-sgRNA-*Gas5* promoted growth of the small intestinal mucosa, elevated the numbers of Paneth cells, and increased the levels of mucosal TJ proteins ZO-1, ZO-2, claudin-1, and claudin-2, associated with an enhancement of gut barrier function. Consistent with the results from the studies in mice, *GAS5* silencing in cultured IECs also increased the abundance of cellular TJ proteins and enhanced epithelial barrier function, as evidenced by an increase in TEER and a decrease in paracellular flux of FITC-dextran in *GAS5*-deficient cells. In contrast, ectopic overexpression of *GAS5* decreased the levels of TJ proteins in primarily cultured intestinal organoids and in cultured IECs, thus disrupting the epithelial barrier function. Together, the results obtained from studies conducted in mice, in organoids, and in cultured cells point to an essential role of *GAS5* in regulating the constitutive renewal of the intestinal mucosal and the epithelial barrier.

The rapid turnover rate of the intestinal epithelium is tightly regulated by many intracellular and extracellular factors (52, 53). Paneth cells are specialized IECs that produce abundant antibacterial proteins and peptides such as lysozyme and Reg3 lectins to protect the epithelium against pathogenic infection (54, 55). Paneth cells are also constituents of the ISC niche located at the base of the crypt, synthesize surface-bound and secreted niche signals, provide metabolic fuel for ISCs, and play an important role in maintaining ISC function and renewal of the intestinal mucosa (54, 55, 56). In other words, our results indicate that *GAS5* regulates the growth of the intestinal epithelium at least partially by altering the function of the Paneth cell/ISC niche. Specific deletion of *Gas5* in IE-Cas9 mice by infection with AAV-sgRNA-*Gas5* increased the numbers of Paneth cells, which are associated with activated ISCs, as indicated by an increase in the levels of OLFM4 staining in the *GAS5*-deficient mucosa. Since ISCs divide daily and are located in a growth factor–rich environment that fully depends on constant secretion of Paneth cells (54, 55), the increased Paneth cells in *Gas5*-KD mice are certain to contribute to ISC activation and subsequent stimulation of the intestinal mucosal growth. In support of these results, we previously reported that targeted deletion of the RBP HuR or lncRNA *uc.173* in mice inhibited renewal of the intestinal epithelium primarily by disrupting the integrity of the Paneth cell/ISC niche (39, 40, 42), while intestinal mucosal tissues from patients with IBD and critical surgical disorders exhibit defects in Paneth cells and ISCs, as evidenced by decreased levels of Paneth cell–derived factors WNT3 and NOTCH2 and LGR5- and OLFM4-positive cells (39).

Our results further indicate that *GAS5* elevates the levels of vtRNAs in IECs and that high levels of *GAS5* decrease TJs and cause epithelial barrier dysfunction by stimulating vtRNA production. vtRNAs are highly conserved across mammalian genomes and expressed in a broad spectrum of eukaryotes (47, 48). Humans express 4 vtRNA paralogs, *vtRNA1-1*, *vtRNA1-2*, *vtRNA1-3*, and *vtRNA2-1*, while mice only produce one vtRNA (47). vtRNAs can be incorporated into giant cytoplasmic ribonucleoprotein (RNP) particles termed vaults, but they also function independently of vault particles; in fact, only approximately 5% of total cellular vtRNA is associated with vaults (57–59). vtRNAs are involved in many cellular processes such as mRNA splicing, nuclear transport, drug resistance, synaptogenesis, lysosome function, apoptosis, influenza virus replication, and tumorigenesis (59–62). The 4 human vtRNAs differ only slightly in their primary and second structures but have distinct pathobiological functions (48). Our recent studies showed that *vtRNA1-1* impairs intestinal epithelial renewal and barrier function by interacting with CUGBP1 (46), while *vtRNA2-1* disrupts gut barrier function by interacting with HuR (49). The present study revealed that *GAS5* is a potent transcriptional enhancer of the *vtRNA-1* gene, since ectopically expressed *GAS5* activated activity of the *vtRNA-1* promoter and increased the levels of cellular vtRNAs, whereas *GAS5* silencing repressed promoter activity and decreased the levels of vtRNAs. Further study revealed that the reduction in TJ levels and the increased paracellular permeability by *GAS5* overexpression were ameliorated by *vtRNA1-1* silencing, demonstrating the importance of vtRNAs in *GAS5*-mediated gut barrier dysfunction.

Establishing how *GAS5* and vtRNA maintains the intestinal epithelium homeostasis may be highly relevant in the clinic. As shown here, human intestinal mucosa with chronic injury/erosion and inflammation from patients with IBD exhibited increased levels of *GAS5*, associated with an increase in mucosal vtRNA abundance, as reported previously (49). EVs isolated from the serum of patients with hemorrhagic shock also displayed increased vtRNAs, including *vtRNA1-1* and *vtRNA2-1*, along with gut barrier dysfunction (46). EVs can transfer bioactive molecules to neighboring or distant tissues with functional impact (63). The involvement of IEC-derived EVs and their cargo ncRNAs, potentially including vtRNAs and *GAS5*, in intestinal epithelium renewal and TJ expression is particularly important in critically ill surgical patients, as they often exhibit widespread, potentially lethal gut barrier dysfunction rather than localized changes in permeability (34, 64). Clearly, more studies are needed to fully elucidate the role of *GAS5* in the regulation of EV vtRNAs in critically ill patients, and to fully define the mechanism by which *GAS5* regulates vtRNA expression in the intestinal epithelium in response to various pathophysiological stresses.

In sum, our findings indicate that *GAS5* affects the homeostasis of the intestinal epithelium by altering the constitutive renewal of the mucosa and the epithelial barrier. Because elevated *GAS5* disrupts gut barrier function and inhibits mucosal growth, at least partially by increasing vtRNA levels, our findings suggest that *GAS5* and vtRNAs, especially those encoded by the *vtRNA-1* locus, are possible therapeutic targets to protect the gut epithelium in critically ill patients.

## Methods

### Sex as a biological variable.

Our studies examined both male and female mice. Sex was not considered as a biological variable in gut barrier function because we did not observe any sex-based differences.

### Generation of IE-Cas9 mice and animal experiments.

To create a loss-of-function mouse model, we used CRISPR/Cas9-knockin mice (41) to specifically delete *Gas5* in the intestinal epithelium by overexpressing sgRNA-*Gas5*. The Cre-dependent Cas9 mouse was purchased from The Jackson Laboratory (strain 026175) and crossed with villin-Cre mice to generate IE-Cas9 mice. As reported previously (42), Cas9 expression in IE-Cas9 mice was restricted to the intestinal epithelium. The specific sgRNA-*Gas5* was selected in HCT116-Cas9 cells for targeting the *Gas5* locus with maximal efficiency before experiments in vivo. AAV-mediated delivery of sgRNA-*Gas5* was constructed and applied in IE-Cas9 mice to direct genome editing of *Gas5*, as described previously (43, 51). Age-matched (male and female) IE-Cas9 mice were injected i.p. with the AAV-sgRNA-*Gas5* to specifically knock out *Gas5* in the intestinal epithelium, while injection with AAV-U6 vector containing no sgRNA (AAV-Con) served as control. The levels of tissue *Gas5* in different organs of IE-Cas9 mice were examined on day 7 after the injection with AAV-sgRNA-*Gas5* or AAV-Con.

Both IE-Cas9 mice and control littermates were housed and handled in a pathogen-free breeding barrier and were cared for by trained technicians and veterinarians. Animals were deprived of food but were allowed free access to tap water for 24 hours before experiments. Two portions of the middle small intestine were taken, one for histological examination and the other for extraction of protein and RNA. The tissues were fixed in formalin and paraffin for immunohistochemical staining, while the mucosa was scraped with a glass slide for various measurements, as described previously (15, 56).

To generate the model of CLP-induced injury, age-matched, male and female mice were anesthetized by Nembutal, and CLP was performed as described previously (36). Forty-eight hours after CLP, two 4-cm segments taken from the middle of the small intestine were removed in each animal, as described previously (15). To generate the colonic mucosal injury model, mice were fed with 3% DSS dissolved in drinking water for 7 consecutive days, as reported previously (23, 35).

### Studies using human tissues.

Human tissue samples were obtained from surplus discarded tissues from the Department of Surgery, University of Maryland Health Science Center (Baltimore, Maryland) and commercial tissue banks (BioIVT). Both men and women (14 women, 15 men), ages 21 years and older, representing different ethnic backgrounds, were included. The mucosal injury/erosions, inflammation, delayed healing, and gut barrier dysfunction were examined as reported previously (15, 39, 40), whereas tissue samples from patients without gut mucosal damage and disrupted barrier served as controls. The mucosal tissues from the colon in patients with UC and from the small intestine in patients with CD were scraped with a glass slide, and total RNA was isolated by using the RNeasy mini kit (Qiagen), as described previously (15, 46).

### Cell and intestinal organoid culture.

HCT116-Cas9 cells were purchased from Genecopoeia and Caco-2 cells were obtained from the American Type Culture Collection; both were maintained under standard culture conditions (23, 39). The culture medium and fetal bovine serum were purchased from Invitrogen and biochemicals were from Sigma-Aldrich. Isolation and culture of primary enterocytes were conducted following the method described previously (22, 65). Briefly, primary crypts were released from the small intestinal mucosa in mice, and the isolated crypts were mixed with matrigel and cultured in IntestiCult organoid growth medium. The levels of DNA synthesis were measured by BrdU incorporation, and the growth of organoids was examined by measuring surface area of organoid horizontal cross sections using the NIS-Elements AR4.30.02 program, as described previously (23).

### Plasmid construction and RNA interference.

An expression vector containing human *GAS5* cDNA under control of the pCMV promoter was constructed and used to increase *GAS5* in intestinal organoids and Caco-2 cells, whereas a vector containing no insert was used as control. Transient transfections were performed using Lipofectamine reagent following the manufacturer’s recommendations (Invitrogen, 116668019); 48 hours after transfection, cells were harvested for analysis. Expression of *GAS5* was silenced by transfection with si-GAS5, as described previously (28, 31). The si-GAS5 and C-siRNA (a scrambled version of si-GAS5) were purchased from Santa Cruz Biotechnology. For each 60-mm cell culture dish, 15 μL of the 20 μM stock duplex si-GAS5 or C-siRNA was used. Forty-eight hours after transfection using Lipofectamine, cells were harvested for analysis. In studies to assay the activity of the *vtRNA1* promoter, the luciferase reporter containing the full-length *vtRNA1* promoter (pGL3 vtRNA1-P) was constructed, as described previously (66). The activity of pGL3 vtRNA1-P was normalized to *Renilla*-driven luciferase activity in every experiment, as described previously (16, 66).

### RT-qPCR and ddPCR analyses.

Total RNA was isolated by using the RNeasy mini kit (Qiagen) and used in RT and PCR amplification reactions, as described previously (67). qPCR analysis was performed using Step-one-plus systems with specific primers, probes, and software (Applied Biosystems). To measure copy numbers of *GAS5*, ddPCR analysis was performed by using QX200 Droplet Digital PCR System (Bio-Rad), as described previously (15). Briefly, PCR reaction mixture containing cDNA was partitioned into aqueous droplets in oil via the QX100 Droplet Generator and then transferred to a 96-well PCR plate. A 2-step thermocycling protocol (95°C for 10 minutes; 40 cycles of [94°C for 30 seconds, 60°C for 60 seconds], 98°C for 10 minutes) was undertaken in a Bio-Rad C1000. The PCR plate was then transferred to the QX100 Droplet Reader for automatic reading of samples in all wells. Copy number of *GAS5* in the intestinal mucosa was directly determined. QuantaSoft 1.7.4 analysis software (Bio-Rad) and Poisson statistics were used to compute droplet concentrations (copies/ng RNA).

### Immunoblotting analysis.

Whole-cell lysates were prepared using 2% SDS, sonicated, and centrifuged at 4°C for 15 minutes, as described previously (13). The supernatants were boiled and size-fractionated by SDS-PAGE. Primary antibodies used were against occludin (Cell Signaling Technology [CST], 91131), claudin-1 (CST, 4933), claudin-2 (CST, 48120), claudin-7 (Invitrogen, 349100), JAM-A (Invitrogen, 361700), ZO-1 (Invitrogen, 339100), ZO-2 (Invitrogen, 374700), E-cadherin (BD Biosciences, 610182), and GAPDH (CST, 2118). The secondary antibodies, anti-rabbit IgG (CST, 7074) and anti-mouse IgG (CST, 7076), were conjugated to horseradish peroxidase. All antibodies utilized in this study were validated for species specificity. Antibody dilutions used for Western blots of occludin, claudin-1, claudin-2, claudin-7, JAM-A, ZO-1, ZO-2, E-cadherin, and GAPDH were 1:800 or 1000 (primary antibody) and 1:2000 (secondary antibody). After the blots were incubated with primary and secondary antibodies, immunocomplexes were developed and visualized using chemiluminescence. Relative protein levels were analyzed by using Bio-Rad Chemidoc and XRS system equipped with Image lab software (version 4.1). We also utilized the “Quantity tool” to determine the band intensity volume; the values were normalized with internal loading control GAPDH.

### Immunofluorescent staining.

The immunofluorescent staining procedure of intestinal mucosal tissues and organoids was carried out as described previously (15, 20). Slides were fixed in 3.7% formaldehyde in PBS and rehydrated. All slides were incubated with primary antibodies against lysozyme (Invitrogen, PA5-89275), DCLK1 (Abcam, ab31704), mucin 2 (Abcam, ab272692), Ki67 (Abcam, ab15580), OLFM4 (CST, 39141), TJs, or E-cadherin in the blocking buffer at concentration of 1:200 or 1:300 dilution at 4ºC overnight and then incubated with secondary antibody conjugated with Alexa Fluor 594 (Molecular Probes) for 2 hours at room temperature. After rinsing 3 times, some slides were incubated with 1 μM DAPI (Electron Microscopy Sciences, 17895) for 10 minutes to stain cell nuclei. Finally, the slides were washed, mounted, and viewed through a Zeiss LSM 710 confocal microscope. Slides were examined in a blinded fashion by coding, and decoding only after examination was completed. Images were processed using Photoshop software (Adobe).

### Measurement of gut permeability.

Epithelial barrier function in vitro was examined by using a 12-mm Transwell plate as described previously (20, 68). FITC-dextran (70 kDa; Sigma-Aldrich), a membrane-impermeable molecule, served as the paracellular tracer and was added at a final concentration of 0.25 mM to the apical bath wells that contained 0.5 mL of medium. The basal bath well had no added tracers and contained 1.5 mL of the same flux assay medium as the apical compartment. All flux assays were performed at 37°C, and the basal medium was collected at different times after addition of FITC-dextran. The concentration of FITC-dextran in the basal medium was determined using a fluorescence plate reader with an excitation wavelength at 490 nm and an emission wavelength of 530 nm. TEER was measured with an epithelial voltmeter under open-circuit conditions (WPI), as described previously (21), and the TEER of all monolayers was normalized to that of control monolayers in the same experiment.

Gut permeability in mice was determined by examining the appearance in blood of FITC-dextran administered by gavage, as described previously (36, 38). Briefly, mice were gavaged with FITC-dextran at a dose of 60 mg/100 g body weight 4 hours before harvest. Blood sample was collected by cardiac puncture. The serum concentration of FITC-dextran was determined using a fluorescence plate reader as described above.

### Statistics.

All values are expressed as mean ± SEM. An unpaired, 2-tailed Student’s *t* test was used when indicated. When assessing multiple groups, 1-way ANOVA was utilized with Tukey’s post hoc test (69). The statistical software used was GraphPad Instat Prism 10. For nonparametric analysis rank comparison, the Kruskal-Wallis test was conducted. A *P* value of less than 0.05 was considered significant

### Study approval.

All animal experiments were performed in accordance with the NIH *Guide for the Care and Use of Laboratory Animals* (National Academies Press, 2011) and were approved by the Institutional Animal Care and Use Committee of University Maryland School of Medicine and Baltimore VA hospital. The human study was approved by the University of Maryland Institutional Review Board.

### Data availability.

All supporting data for each figure panel are available in the Supporting Data Values file. Any additional information required to reanalyze the data reported in this paper is available upon request.

## Author contributions

TXY performed most experiments and summarized data. HKC, AV, BW, HZ, and ASGC participated in experiments using mice, immunoprecipitation assays, and experiments conducted in intestinal organoids and cultured IECs. RK, MG, and LX participated in experiments using human tissues, data analysis, and edited the manuscript. JYW designed experiments, analyzed data, prepared figures, and drafted the manuscript. All authors reviewed the final manuscript.

## Funding support

This work is the result of NIH funding, in whole or in part, and is subject to the NIH Public Access Policy. Through acceptance of this federal funding, the NIH has been given a right to make the work publicly available in PubMed Central.

- NIH grants DK57819 and DK68491 (to JYW).
- NIH grant AG084613 (to LX).
- NIH grant T32DK67872 (to AV).
- National Institute on Aging Intramural Research Program/NIH grant AG000511 (to MG).
- US Department of Veterans Affairs Merit Review Award (to JYW).

## Supplementary Material

Supplemental data

Unedited blot and gel images

Supporting data values

## Version 1 01/22/2026

In-Press Preview

## Version 2 03/09/2026

Electronic publication

## Version 3 03/10/2026

Copyeditor query removed

## Version 4 03/10/2026

inadvertent italicized text corrected

## References

[1] Ferreira-Gonzalez S (2025). Senescence, aging and disease throughout the gastrointestinal system. Gastroenterology.

[2] Ren X (2024). DHX9 maintains epithelial homeostasis by restraining R-loop-mediated genomic instability in intestinal stem cells. Nat Commun.

[3] Rao JN (2020). Polyamines in gut epithelial renewal and barrier function. Physiology (Bethesda).

[4] Horowitz A (2023). Paracellular permeability and tight junction regulation in gut health and disease. Nat Rev Gastroenterol Hepatol.

[5] Camilleri M (2019). Leaky gut: mechanisms, measurement and clinical implications in humans. Gut.

[6] Lessey LR (2022). Adherens junction proteins on the move-from the membrane to the nucleus in intestinal diseases. Front Cell Dev Biol.

[7] Villablanca EJ (2022). Mechanisms of mucosal healing: treating inflammatory bowel disease without immunosuppression?. Nat Rev Gastroenterol Hepatol.

[8] Assimakopoulos SF (2018). Gut-origin sepsis in the critically ill patient: pathophysiology and treatment. Infection.

[9] Ciorba MA (2024). Challenges in IBD research 2024: preclinical human IBD mechanisms. Inflamm Bowel Dis.

[10] Ponting CP (2009). Evolution and functions of long noncoding RNAs. Cell.

[11] Herman AB (2022). Integrated lncRNA function upon genomic and epigenomic regulation. Mol Cell.

[12] Xiao L (2019). Long noncoding RNAs in intestinal epithelium homeostasis. Am J Physiol Cell Physiol.

[13] Xiao L (2016). Long noncoding RNA SPRY4-IT1 regulates intestinal epithelial barrier function by modulating the expression levels of tight junction proteins. Mol Biol Cell.

[14] Wang M (2025). Gut microbiota protects against colorectal tumorigenesis through lncRNA SNHG9. Dev Cell.

[15] Xiao L (2021). Circular RNA CircHIPK3 promotes homeostasis of the intestinal epithelium by reducing microRNA 29b function. Gastroenterology.

[16] Xiao L (0185). Long noncoding RNA uc.173 promotes renewal of the intestinal mucosa by inducing degradation of microRNA 195. Gastroenterology.

[17] Li XX (2020). Interaction between HuR and *circPABPN1* modulates autophagy in the intestinal epithelium by altering ATG16L1 translation. Mol Cell Biol.

[18] Yoon JH (2012). LincRNA-p21 suppresses target mRNA translation. Mol Cell.

[19] Cairns CA (2024). Posttranscriptional regulation of intestinal mucosal growth and adaptation by noncoding RNAs in critical surgical disorders. J Invest Surg.

[20] Yu TX (2020). Long noncoding RNA H19 impairs the intestinal barrier by suppressing autophagy and lowering paneth and goblet cell function. Cell Mol Gastroenterol Hepatol.

[21] Zou T (2016). H19 long noncoding RNA regulates intestinal epithelial barrier function via microRNA 675 by interacting with RNA-binding protein HuR. Mol Cell Biol.

[22] Wang JY (2018). Regulation of intestinal epithelial barrier function by long noncoding RNA *uc.173* through interaction with microRNA 29b. Mol Cell Biol.

[23] Yu TX (2022). Long noncoding RNA uc.230/CUG-binding protein 1 axis sustains intestinal epithelial homeostasis and response to tissue injury. JCI Insight.

[24] Tang R (2017). The long non-coding RNA GAS5 regulates transforming growth factor β (TGF-β)-induced smooth muscle cell differentiation via RNA Smad-binding elements. J Biol Chem.

[25] Schneider C (1988). Genes specifically expressed at growth arrest of mammalian cells. Cell.

[26] Lin G (2022). Research progress of long non-coding RNA GAS5 in malignant tumors. Front Oncol.

[27] Filippova EA (2021). Long noncoding RNA GAS5 in breast cancer: epigenetic mechanisms and biological functions. Int J Mol Sci.

[28] Zhao H (2018). Lowly-expressed lncRNA GAS5 facilitates progression of ovarian cancer through targeting miR-196-5p and thereby regulating HOXA5. Gynecol Oncol.

[29] Kino T (2010). Noncoding RNA GAS5 is a growth arrest- and starvation-associated repressor of the glucocorticoid receptor. Sci Signal.

[30] Patel N (2019). LncRNA GAS5 directed therapeutic increases insulin receptor expression in adipocytes. J Endocr Soc.

[31] Larrasa-Alonso J (2021). The SRSF4-GAS5-glucocorticoid receptor axis regulates ventricular hypertrophy. Circ Res.

[32] Ling H (2021). LncRNA GAS5 inhibits miR-579-3p to activate SIRT1/PGC-1α/Nrf2 signaling pathway to reduce cell pyroptosis in sepsis-associated renal injury. Am J Physiol Cell Physiol.

[33] Sang L (2021). Mitochondrial long non-coding RNA GAS5 tunes TCA metabolism in response to nutrient stress. Nat Metab.

[34] Zhou Q (2025). Human colonic EVs induce murine enteric neuroplasticity via the lncRNA GAS5/miR-23/NMDA NR2B axis. JCI Insight.

[35] Chassaing B (2014). Dextran sulfate sodium (DSS)-induced colitis in mice. Curr Protoc Immunol.

[36] Hubbard WJ (2005). Cecal ligation and puncture. Shock.

[37] Liu L (2017). HuR enhances early restitution of the intestinal epithelium by increasing Cdc42 translation. Mol Cell Biol.

[38] Yu TX (2011). Chk2-dependent HuR phosphorylation regulates occludin mRNA translation and epithelial barrier function. Nucl Acid Res.

[39] Xiao L (2023). Control of Paneth cell function by HuR regulates gut mucosal growth by altering stem cell activity. Life Sci Alliance.

[40] Xiao L (2019). RNA-binding protein HuR regulates paneth cell function by altering membrane localization of TLR2 via post-transcriptional control of CNPY3. Gastroenterology.

[41] Platt RJ (2014). CRISPR-Cas9 knockin mice for genome editing and cancer modeling. Cell.

[42] Xiao L (2026). Long noncoding RNA uc173 is a novel regulator of mitochondrial metabolism driving intestinal mucosal growth. Cell Mol Gastroenterol Hepatol.

[43] You H (2021). CRISPR/Cas9-mediated genome editing of Schistosoma mansoni acetylcholinesterase. FASEB J.

[44] Guo X (2005). Polyamines are necessary for synthesis and stability of occludin protein in intestinal epithelial cells. Am J Physiol Gastrointest Liver Physiol.

[45] Zhang T (2020). Early downregulation of P-glycoprotein facilitates bacterial attachment to intestinal epithelial cells and thereby triggers barrier dysfunction in a rodent model of total parenteral nutrition. FASEB J.

[46] Sharma S (2025). Noncoding vault RNA1-1 impairs intestinal epithelial renewal and barrier function by interacting with CUG-binding protein 1. Cell Mol Gastroenterol Hepatol.

[47] Stadler PF (2009). Evolution of vault RNAs. Mol Biol Evol.

[48] Jouravleva K, Zamore PD (2025). A guide to the biogenesis and functions of endogenous small non-coding RNAs in animals. Nat Rev Mol Cell Biol.

[49] Ma XX (2023). Small noncoding vault RNA2-1 disrupts gut epithelial barrier function via interaction with HuR. EMBO Rep.

[50] Frank F (2020). The lncRNA growth arrest specific 5 regulates cell survival via distinct structural modules with independent functions. Cell Rep.

[51] Seidel E (2021). Enhanced Ca^2+^ signaling, mild primary aldosteronism, and hypertension in a familial hyperaldosteronism mouse model (*Cacna1h*^M1560V/+^). Proc Natl Acad Sci U S A.

[52] Sharma S (2023). HuR and its interactions with noncoding RNAs in gut epithelium homeostasis and diseases. Front Biosci (Landmark Ed).

[53] Bankaitis ED (2018). Reserve stem cells in intestinal homeostasis and injury. Gastroenterology.

[54] Quintero M, Samuelson LC (2025). Paneth cells: dispensable yet irreplaceable for the intestinal stem cell niche. Cell Mol Gastroenterol Hepatol.

[55] Yilmaz OH (2012). mTORC1 in the Paneth cell niche couples intestinal stem-cell function to calorie intake. Nature.

[56] Xiao L (2013). miR-29b represses intestinal mucosal growth by inhibiting translation of cyclin-dependent kinase 2. Mol Biol Cell.

[57] Amort M (2015). Expression of the vault RNA protects cells from undergoing apoptosis. Nat Commun.

[58] Horos R (2019). The small non-coding vault RNA1-1 acts as a riboregulator of autophagy. Cell.

[59] Kickhoefer VA (1998). Vaults are up-regulated in multidrug-resistant cancer cell lines. J Biol Chem.

[60] Stok JE (2025). Vault RNAs aid RNA virus infection by facilitating cytoplasmic localization of hnRNP C and ELAVL1. Cell Rep.

[61] Wakatsuki S, Araki T (2021). Novel molecular basis for synapse formation: small non-coding vault RNA functions as a riboregulator of MEK1 to modulate synaptogenesis. Front Mol Neurosci.

[62] Ferro I (2022). The human vault RNA enhances tumorigenesis and chemoresistance through the lysosome in hepatocellular carcinoma. Autophagy.

[63] Das S (2019). The extracellular RNA communication consortium: establishing foundational knowledge and technologies for extracellular RNA research. Cell.

[64] Carter SR (2013). Intestinal barrier disruption as a cause of mortality in combined radiation and burn injury. Shock.

[65] Yu S (2020). Paneth cell-derived lysozyme defines the composition of mucolytic microbiota and the inflammatory tone of the intestine. Immunity.

[66] Liu L (2006). Polyamine-modulated c-Myc expression in normal intestinal epithelial cells regulates p21Cip1 transcription through a proximal promoter region. Biochem J.

[67] Chung HK (2015). Transgenic expression of miR-222 disrupts intestinal epithelial regeneration by targeting multiple genes including frizzled-7. Mol Med.

[68] Bischoff SC (2014). Intestinal permeability--a new target for disease prevention and therapy. BMC Gastroenterol.

[69] Harter HL (1960). Critical values for Duncan’s new multiple range test. Biometrics.

