# Supplementary material for: Long noncoding RNA GAS5 disrupts intestinal epithelial barrier function by increasing small vault RNA levels
Source: JCI Insight. 2026 Jan 22;11(5):e198593. doi: 10.1172/jci.insight.198593 (PMC13041674; doi:10.1172/jci.insight.198593)
Supplement: Supplemental data [file jciinsight-11-198593-s290.pdf]

A

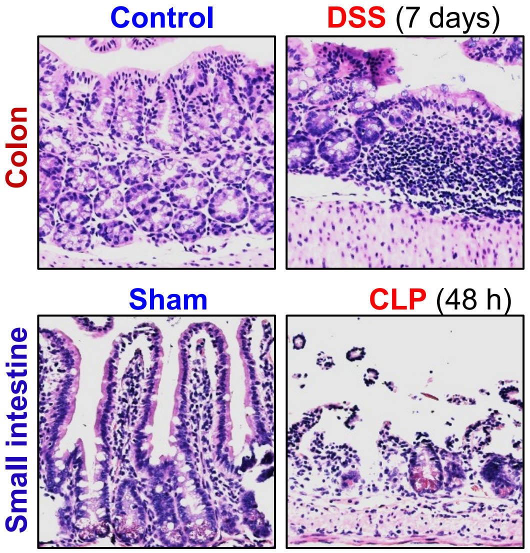

B

sgRNA #1: **GAGTTGCGGGCGCACAGGTG**
Protospacer adjacent motif (PAM) **AGG**

sgRNA #2: **GCCCAGTGACTGGATGGTAT** PAM **TGG**

sgRNA #3: **CCCCGCGGCAAGGGAGTTGC** PAM **GGG**

sgRNA #4: **ACCAATACCATCCAGTCACT** PAM **GGG**

C

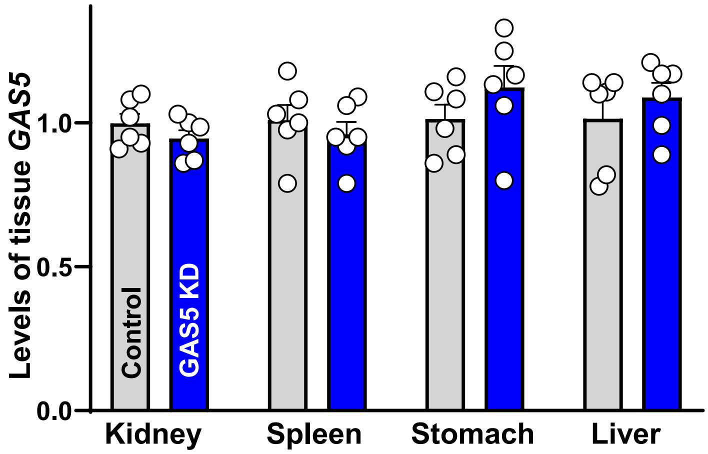

Supplemental Figure 1. (**A**) Images of H&E staining of mouse DSS-induced colitis (top) and CLP-induced small intestinal mucosal injury (bottom). (**B**) Sequences of sgRNAs used for deleting *GAS5* in IE-Cas9 mice. (**C**) Changes in the levels of *GAS5* in kidney, spleen, stomach, and liver in control and GAS5 KD mice. Values are the means ± SEM (n = 6).

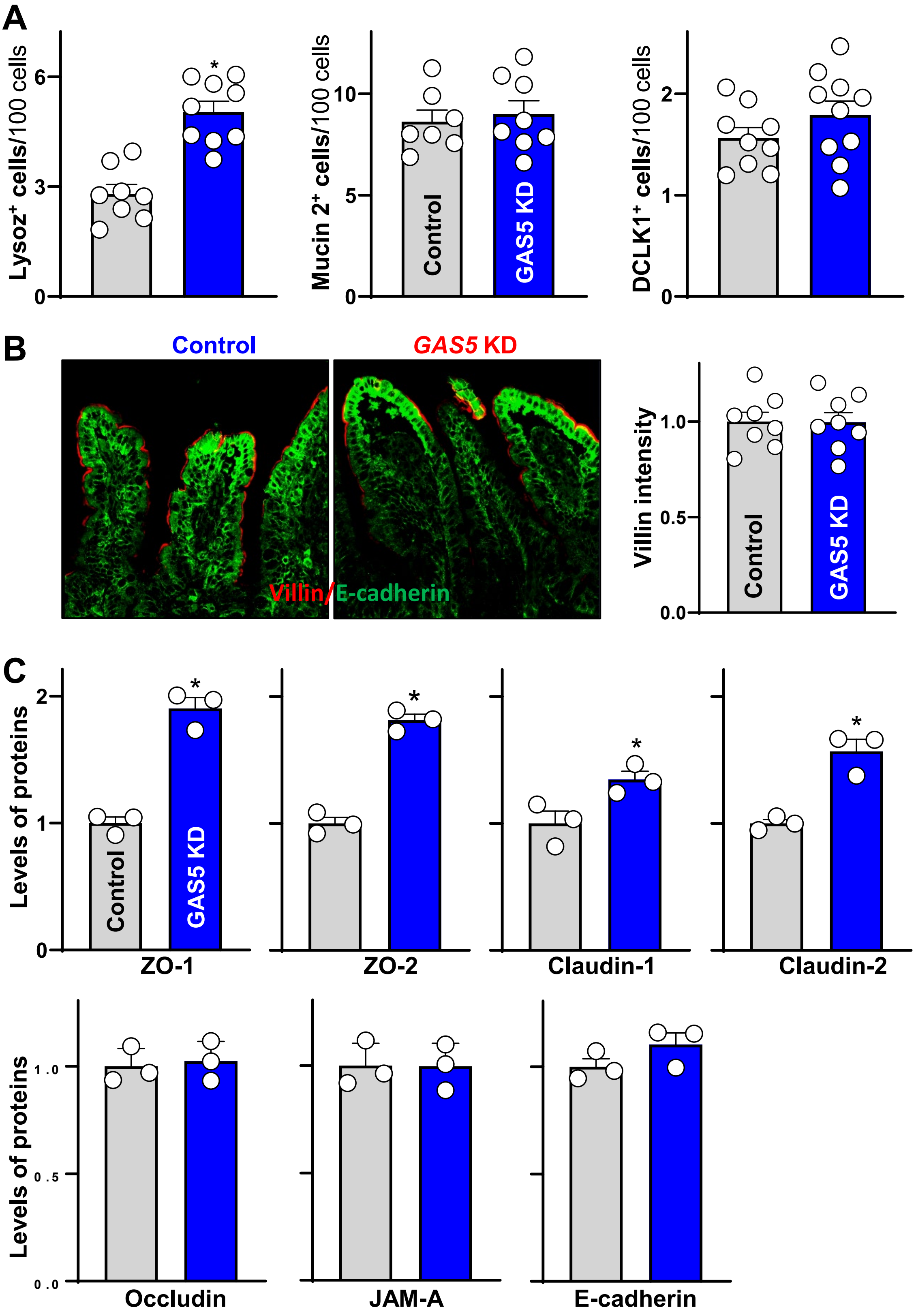

Supplemental Figure 2. (**A**) Changes in lysozyme (lysoz)-positive, mucin 2-positive, and DCLK1-positive cells in the small intestinal mucosa as described in Figure 3A. Values are the means ± SEM (*n* = 8 or 10). **P* < 0.05 compared with controls. (**B**) Enterocyte differentiation in small intestinal mucosa of control and GAS KD mice as measured by villin immunostaining assay. Values are the means ± SEM (*n* = 8). (**C**) Densitometric analysis of immunoblots shown in Figure. 3B. Values are the means ± SEM (*n* = 3). * *P* < 0.05 compared with controls.

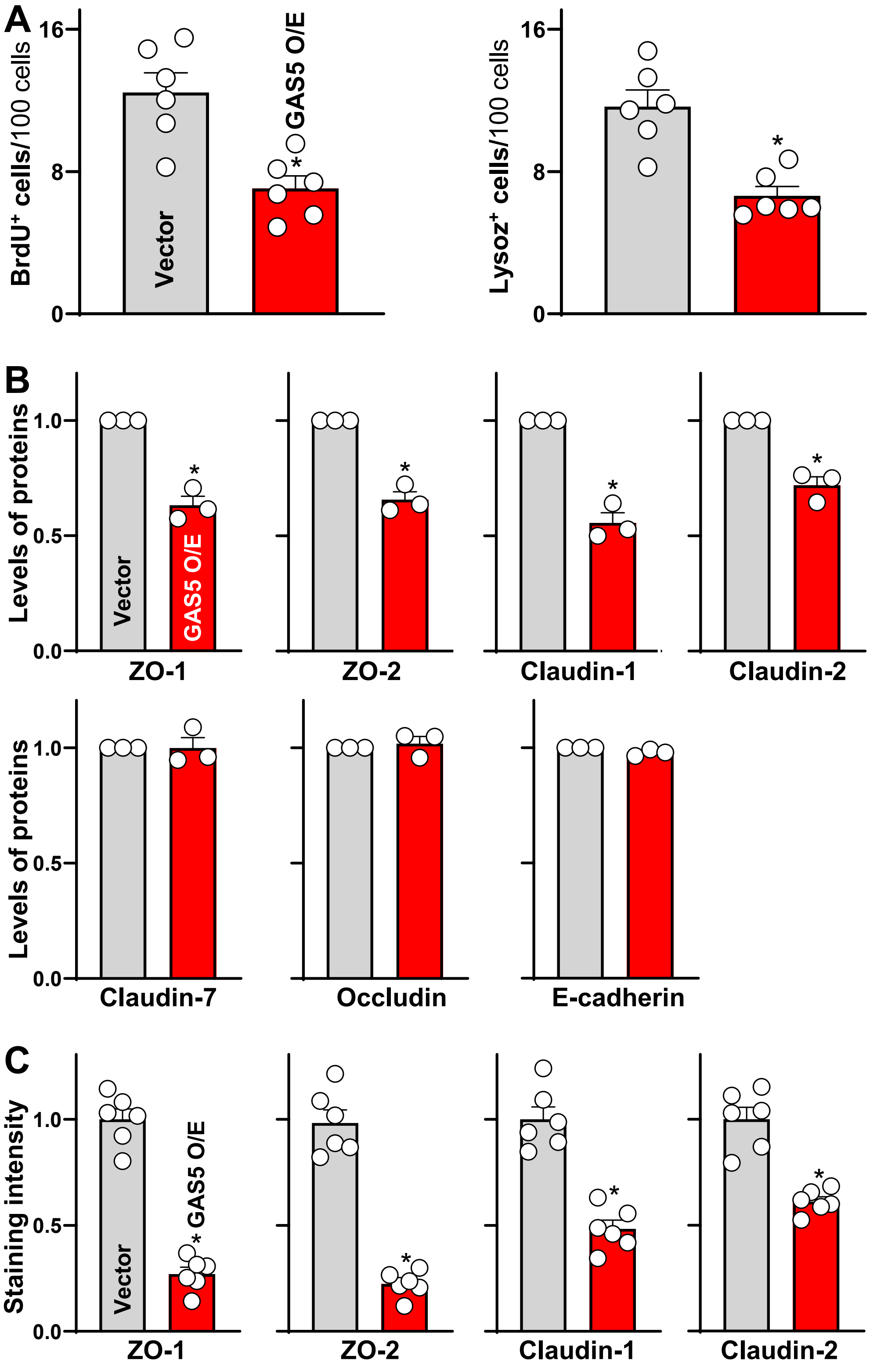

Supplemental Figure 3. (**A**) Changes in the levels of BrdU-positive (*left*) and lysozyme (lysoz)-positive (*right*) cells in intestinal organoids transfected with control vector and GAS5 expression vector (GAS5 O/E) as described in Figure 4C&D. Values are the means ± SEM (n = 6). * $P < 0.05$ compared with control vector. (**B**) Densitometric analysis of immunoblots shown in Figure 4E. *$P < 0.05$ compared with controls (n = 3). (**C**) Fluorescence staining intensity of various TJ proteins described in in Figure 4F. * $P < 0.05$ compared with controls (n = 6).

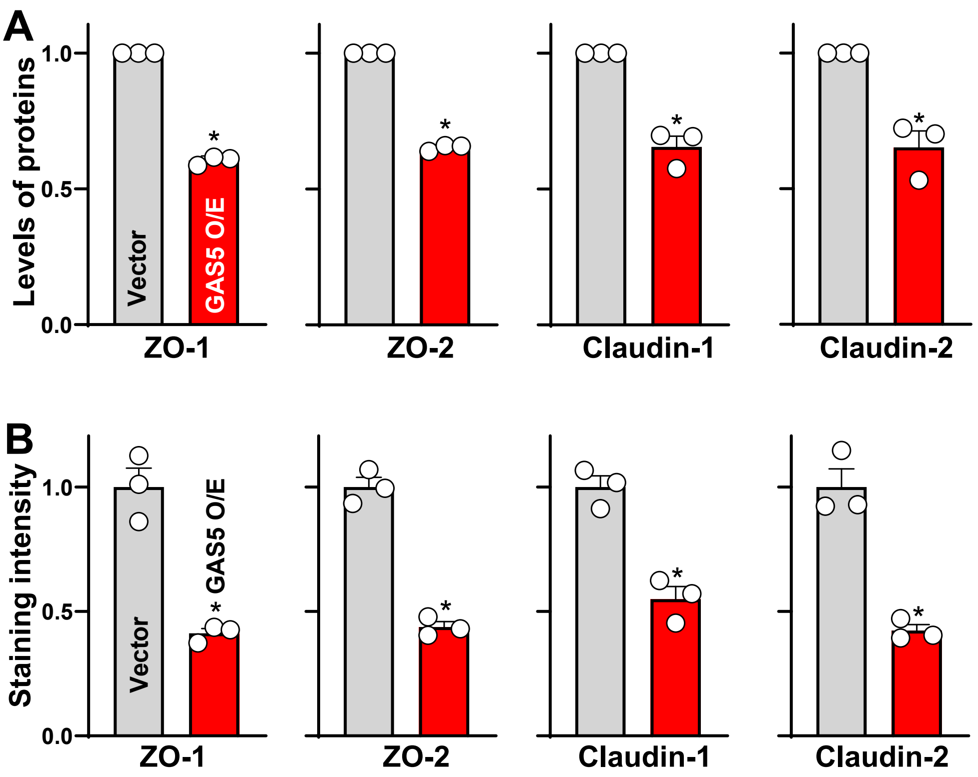

Supplemental Figure 4. (**A**) Densitometric analysis of immunoblots of various tight junction proteins in Caco-2 cells after GAS5 overexpression (GAS5 O/E) as shown in Figure 5B. Values are the means ± SEM (*n* = 3). * *P* < 0.05 compared with control vector. (**B**) Fluorescence intensity of various tight junction proteins as described in Figure 5C. * *P* < 0.05 compared with controls (*n* = 3).

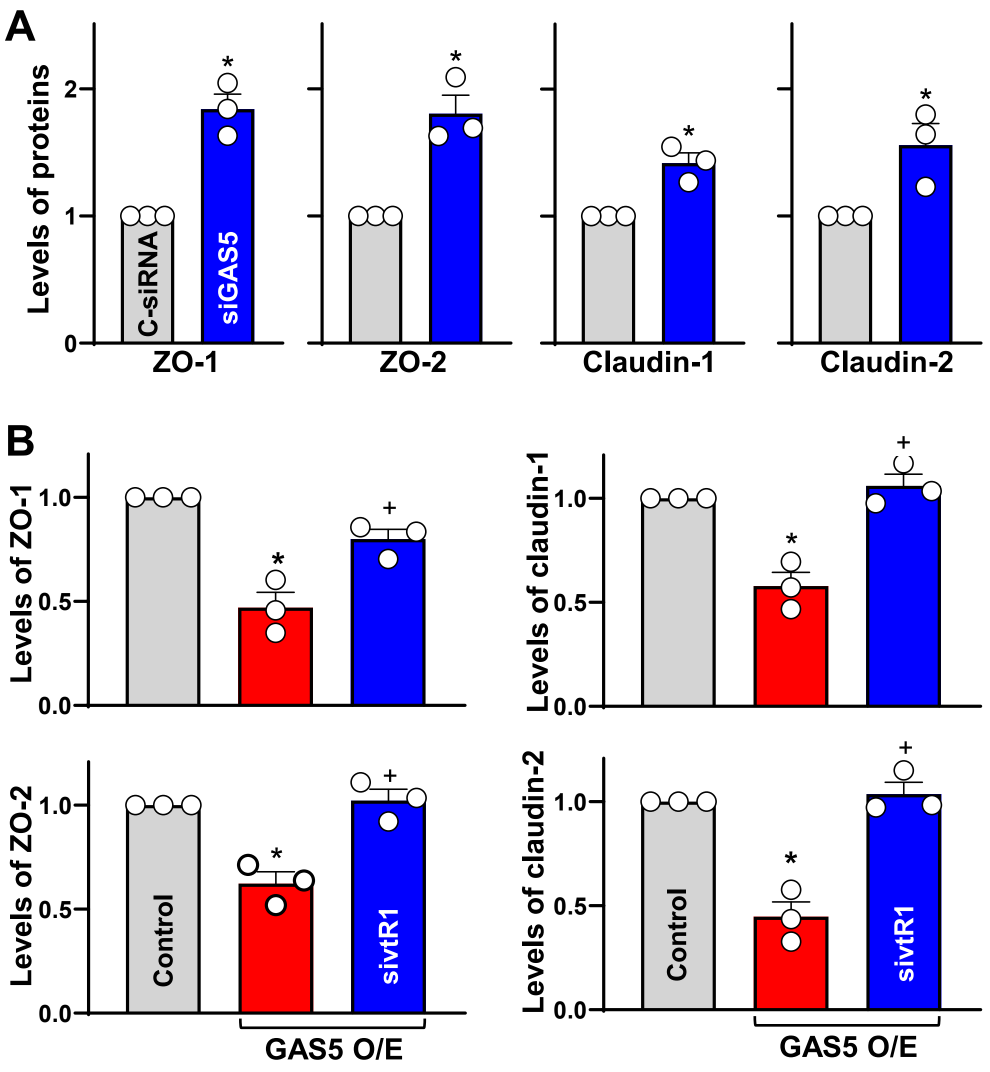

Supplemental Figure 5. (**A**) Densitometric analysis of immunoblots of various tight junction protein in Caco-2 cells after GAS5 silencing as shown in Figure 6B. Values are the means ± SEM (n = 3). * $P < 0.05$ compared with C-siRNA. (**B**) Densitometric analysis of immunoblots of tight junction proteins 48 h after transfection with GAS5 expression vector alone or co-transfection with GAS5 expression vector and siRNA targeting vtRNA1 (sivtR1) as shown in Figure 8B. $^{*,+}$ $P < 0.05$ compared with controls and GAS5 O/E, respectively (n = 3).
