# Supplementary material for: Long noncoding RNA GAS5 disrupts intestinal epithelial barrier function by increasing small vault RNA levels
Source: JCI Insight. 2026 Jan 22;11(5):e198593. doi: 10.1172/jci.insight.198593 (PMC13041674; doi:10.1172/jci.insight.198593)

Figure 3B-left

Figure 3B-right

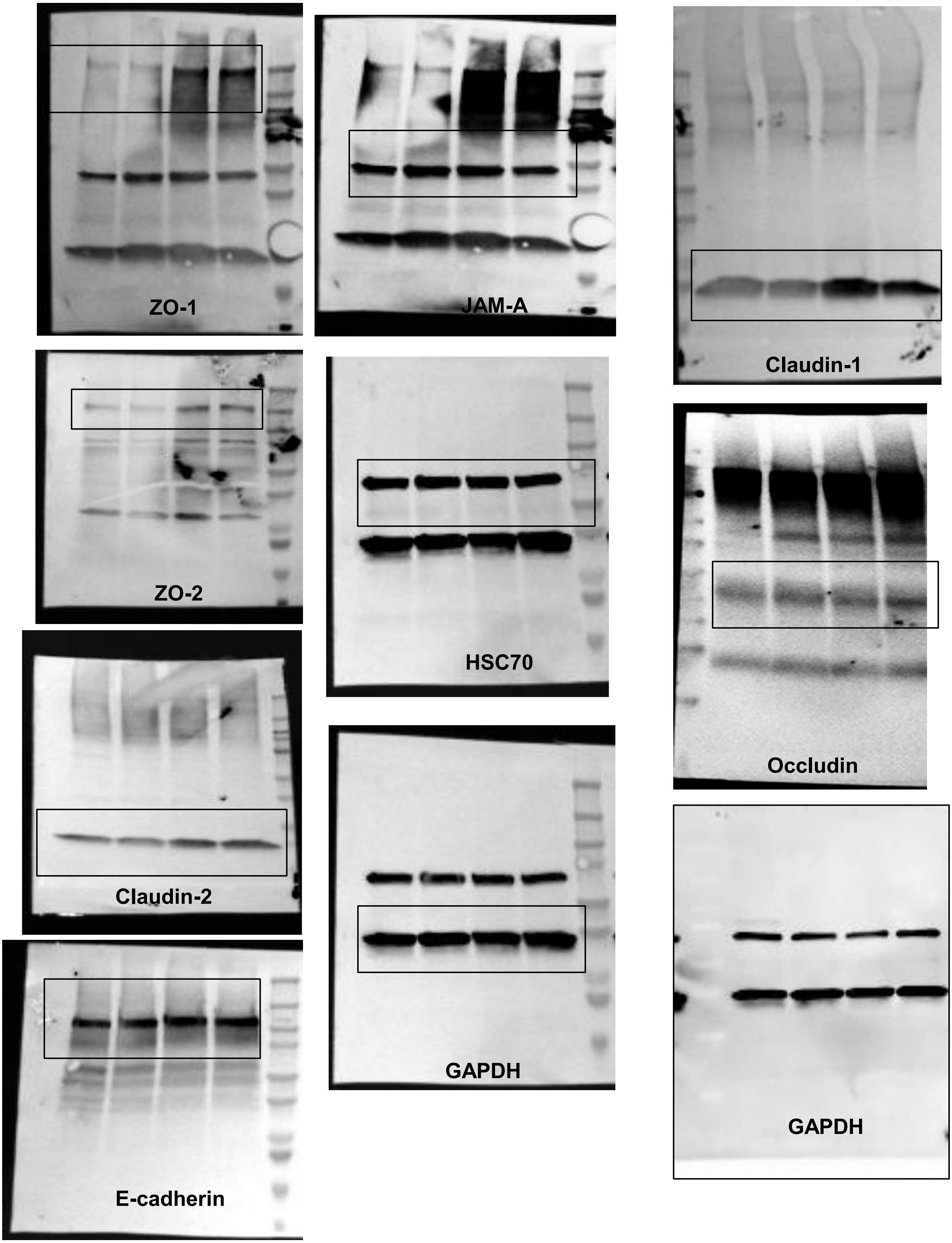

- ZO-1 and JAM-A was from same immunoblot incubated with both anti-ZO-1 and anti-JAM-A antibodies together, while HSC70 and GAPDH was from a immunoblot incubated with both anti-HSC70 and anti-GAPDH antibodies together.

Figure 4E

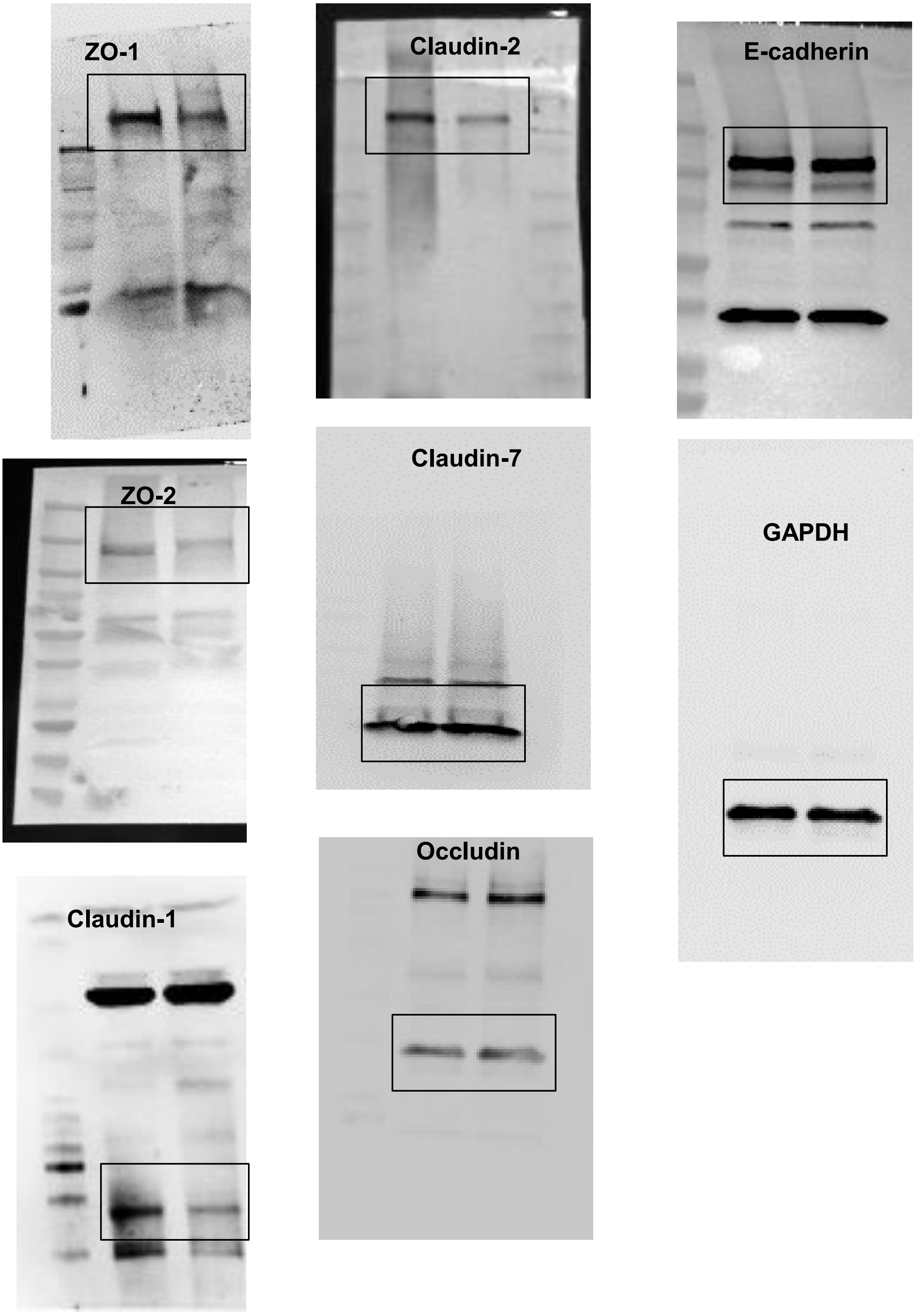

ZO-1
Claudin-2
E-cadherin
ZO-2
Claudin-7
GAPDH
Claudin-1
Occludin

Figure 5B-Top

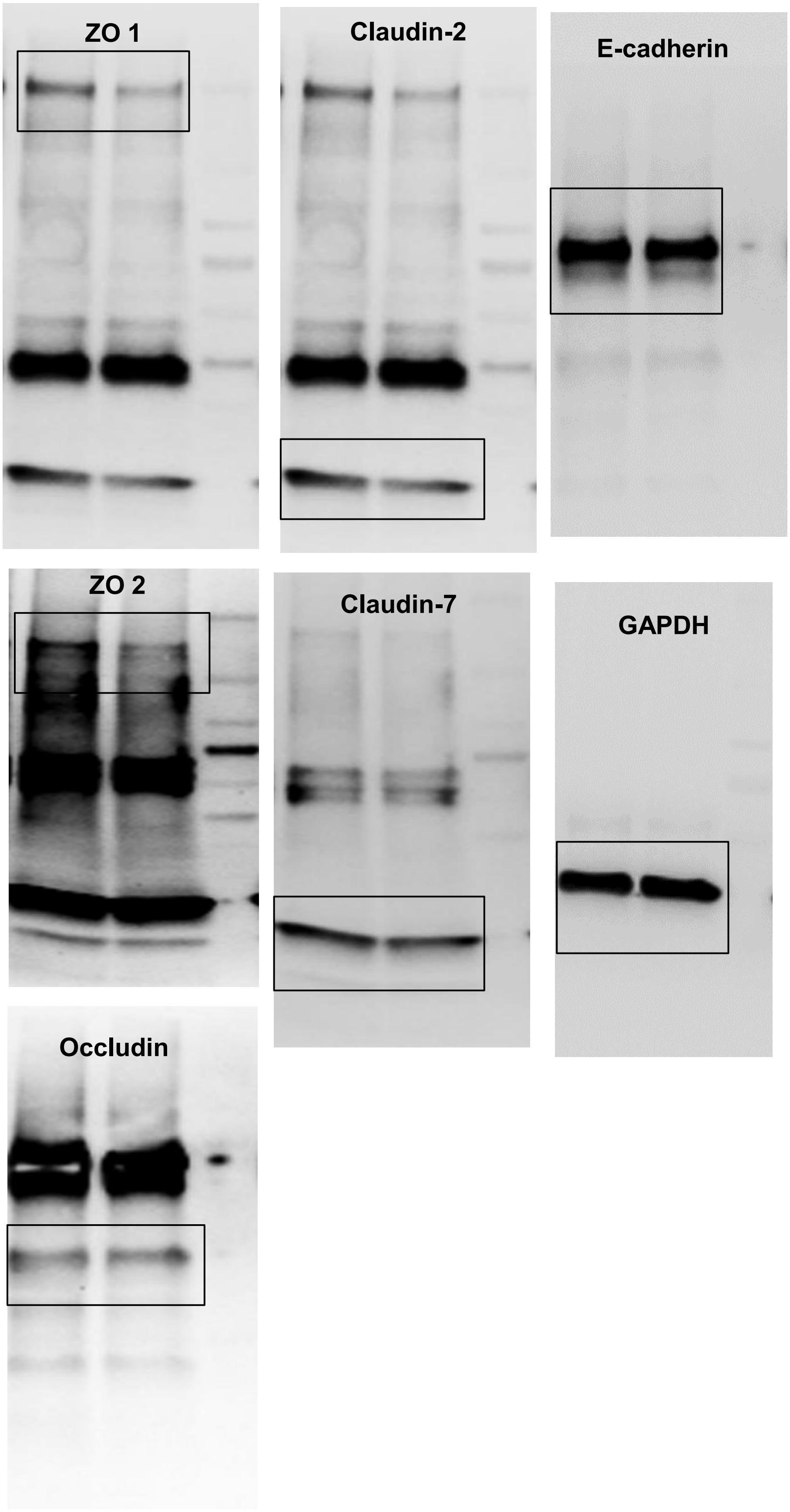

Figure 5B-low

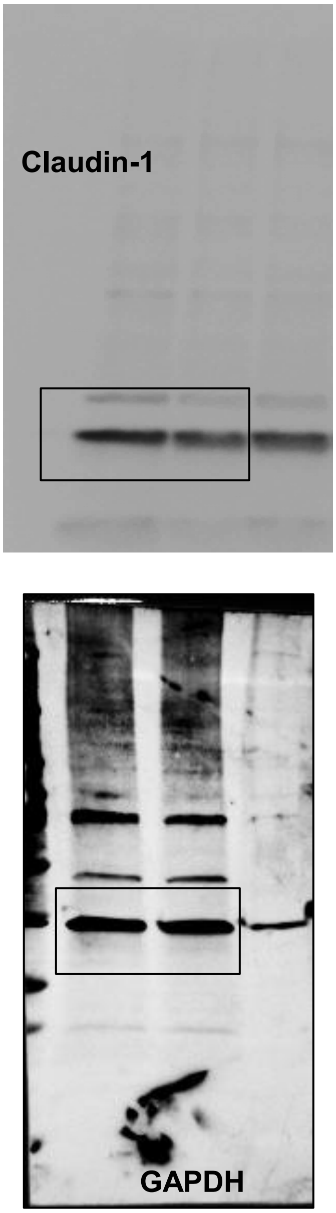

- ZO-1 and claudin-2 was from same immunoblot incubated with both anti-ZO-1 and anti-claudin-2 antibodies together.

Figure 6B-Top

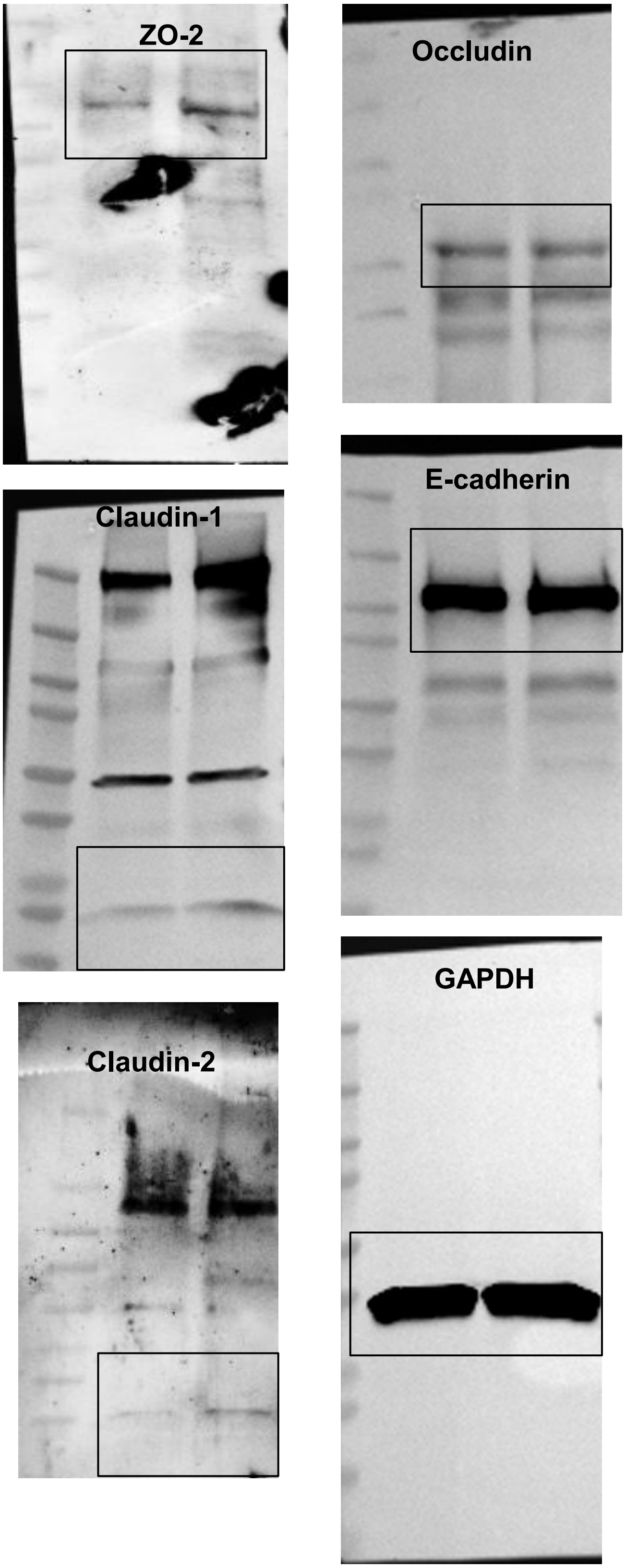

Figure 6B-Low

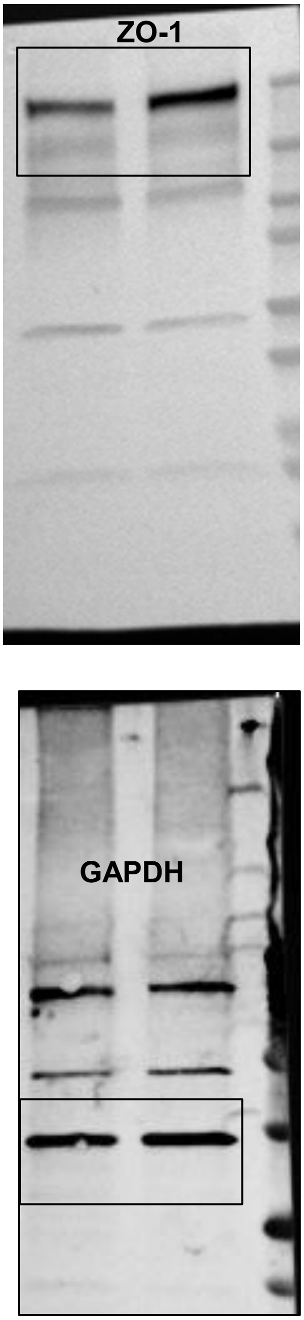

Figure 8B

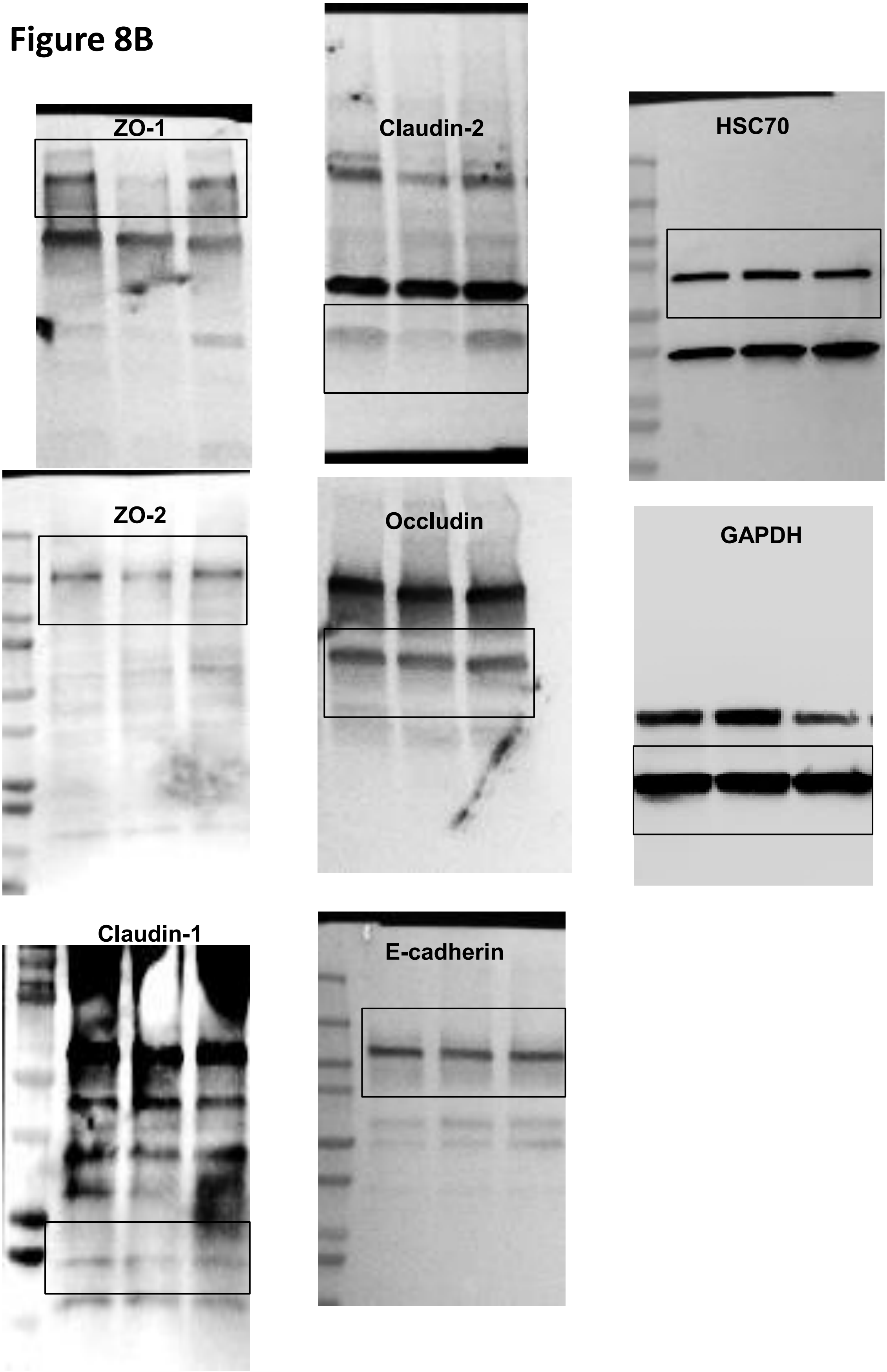

Supplement: Unedited blot and gel images [file jciinsight-11-198593-s291.pdf]
